# Label‐Free Electrochemical Impedance Spectroscopy for Biosensing: Evolving Interfaces and Mechanistic Insights

**DOI:** 10.1002/smsc.202500380

**Published:** 2025-10-21

**Authors:** Nandhinee Radha Shanmugam, Joshua Rainbow, Jeong‐Chan Lee, Pawan Jolly, Donald E. Ingber

**Affiliations:** ^1^ Wyss Institute for Biologically Inspired Engineering Harvard University Boston Massachusetts 02115 USA; ^2^ Center for Advanced Molecular Recognition Korea Institute of Science and Technology Seoul 02792 South Korea; ^3^ Harvard John A. Paulson School of Engineering and Applied Sciences Cambridge Massachusetts 02139 USA; ^4^ Vascular Biology Program and Department of Surgery Boston Children's Hospital and Harvard Medical School Boston Massachusetts 02115 USA

**Keywords:** biosensors, Debye length, electrochemical impedance spectroscopy, electrode/electrolyte interfaces, label‐free, surface modifications

## Abstract

The evolution of label‐free electrochemical biosensors has revolutionized the field of analytical detection by enabling rapid, direct, and sensitive detection of a wide range of analytes. Electrochemical impedance spectroscopy (EIS) provides mechanistic insight into the interfacial changes occurring at the electrode/electrolyte interface, thereby enabling real‐time monitoring. Direct detection of molecular binding events at the electrode interface is made possible by sensing measurable shifts in interfacial impedance characteristics. Despite their versatility, the commercial translation of EIS‐enabled biosensors has been hindered by challenges in achieving robust sensitivity, specificity, and reproducibility. Recent progress in the field, including integration of nanoengineered electrode materials and novel biorecognition elements, has addressed some of these limitations, resulting in marked improvements in EIS‐based biosensor performance. This review discusses the mechanistic principles underlying label‐free EIS biosensing and highlights recent developments in electrode surface modification and sensor architecture. It also explores the integration of novel biorecognition elements and describes how their impact on sensor performance may be assessed. Current limitations and future directions for the application of EIS‐enabled sensors in clinical diagnostics, environmental analysis, and food safety monitoring are also considered.

## Graphical abstract

Electrochemical impedance spectroscopy‐based label‐free biosensors offer rapid, direct, and sensitive analyte detection by measuring impedance changes in real time. Innovations in nanoengineered electrode surfaces and biorecognition elements have enhanced their performance facilitating point‐of‐care applications, despite ongoing challenges in robustness and reproducibility. This review highlights mechanistic principles, novel sensor architectures, and their applications in clinical, environmental, and food safety monitoring.

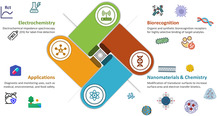

## 1 Introduction

Biosensors are integrated analytical devices that combine biorecognition elements, such as antibodies, aptamers, enzymes, cells, or nucleic acids, with a physicochemical transducer to convert specific biological binding interactions into quantifiable signals.^[^ ^1^ ^]^ At its core lies the biorecognition element, which serves as a highly selective capture probe designed to bind with high affinity and specificity to the target analyte. The physicochemical changes induced by these binding events manifest as optical, electrical, or mechanical signal responses depending on the chosen transduction method.^[^ ^1^, ^2^ ^]^ Electrochemical transduction has garnered significant attention due to its inherent simplicity, versatility, ease of miniaturization, simple device integration, and cost‐effectiveness.^[^ ^2^ ^]^ Electrochemical biosensors detect changes in electrical properties, such as electric current, potential, or impedance, that alter as a result of charge perturbation at the electrode surface arising from ion or electron transfer (accumulation or depletion) in response to analyte binding.^[^ ^3^ ^]^ Voltammetric, amperometric, conductometric, or impedimetric techniques are employed to measure these changes, each exploiting a specific electrical property to gain insights into the biorecognition events and determine analyte concentration.^[^ ^3^, ^4^ ^]^

In 1962, Clark and Lyons laid the foundation for the field of electrochemical sensors with the first demonstration of a glucose enzyme‐based electrode.^[^ ^5^ ^]^ Since that time, there has been innovative development and evolution of multiple generations of biosensors, which ultimately led to the creation of the glucometer—an amperometric‐based biosensor which has been successfully commercialized and used by millions of diabetic patients worldwide to monitor their blood glucose levels on a day‐to‐day basis. Despite tremendous research efforts aimed at expanding the application of electrochemical biosensors for other biomolecular analytes, they are not in wide use. The question remains: why have these sensors for analytes besides glucose not achieved similar market penetration? While recent advances in micro‐ and nanofabrication as well as microfluidics have made possible miniaturization enabling multiplexed detection, low sample volume, and seamless integration with electronic readers, development of these biosensors remains largely confined to academic and industrial laboratories. This limitation is mainly attributed to the loss of biosensor performance in complex biological samples, where interferences from other biomolecules passivate sensor surfaces, resulting in electrode fouling, which affects sensor sensitivity, specificity, and accuracy.^[^ ^6^ ^]^ The lack of robust and selective biorecognition elements for many analytes also further impacts the sensor reliability and shelf life.

Engineered nanostructured electrode surfaces or the integration of antifouling coatings, such as polymer nanocomposites,^[^ ^7^ ^]^ hydrogels,^[^ ^8^ ^]^ and zwitterionic peptides^[^ ^9^ ^]^ have been shown to reduce nonspecific interactions and improve signal‐to‐noise ratio in these biosensors enabling detection in complex sample matrices but persistent challenges remain.^[^ ^6^ ^]^ Signal detection in electrochemical biosensors can be classified into either label‐based (indirect) or label‐free (direct) approaches, which in turn determine the complexity of the biomolecular assay, turnaround time, and overall workflow of the biosensing process.^[^ ^10^ ^]^ Indirect biosensors utilize labels (e.g., an enzyme, fluorophore, and redox‐active molecule) to detect analyte binding. The label participates in a secondary reaction, often catalytic, that produces electroactive species facilitating electron transfer at the electrode/electrolyte interface, resulting in measurable electrochemical signal changes that correlates with analyte concentration.^[^ ^11^ ^]^ While this approach provides high sensitivity, it often involves complex, multistep assay protocols which are laborious, expensive, and time‐consuming. Although labels amplify the signal response by enzyme or redox coupling, they also may alter native properties or binding kinetics, further limiting their use in real‐time monitoring applications.

In contrast, label‐free direct biosensors rely on electrochemical signal responses arising directly from analyte binding, thereby simplifying assay design and offering rapid response capabilities. For example, affinity‐based label‐free biosensors detect changes in interfacial properties resulting from the formation of antibody–antigen immunocomplexes, which may yield valuable insights into the kinetic and mechanistic behaviors underlying this biorecognition event.^[^ ^3^, ^11^ ^]^ This direct approach eliminates the need for secondary detection agents, reduces assay complexity, and preserves the intrinsic characteristics of biomolecules. Electrochemical impedance spectroscopy (EIS), an extremely sensitive and powerful electrochemical technique, can be used to detect these physicochemical changes occurring at the electrode/electrolyte interface by monitoring impedance responses.

Although EIS‐enabled direct biosensors offer a simple and straightforward approach to analyte detection, the development of robust, user‐friendly, and cost‐effective sensing platforms, as well as their practical implementation, remains challenging. A noteworthy limitation commonly observed in these biosensors is their relatively low sensitivity. Upon analyte binding, changes in interfacial properties are often subtle, which can further be diminished by the Debye length screening due to mobile ions in high ionic solutions, including physiological buffers.^[^ ^12^ ^]^

The Debye length determines the thickness of the electrical double layer (EDL) at the electrode/electrolyte interface, which is the key interfacial property that forms the basis for the impedance response in label‐free EIS‐based biosensing. For optimal detection of specific binding events, it is essential that these events occur within or very close to the Debye screening layer, which is intrinsically small (≈1–2 nm) compared to larger biorecognition elements like antibodies (10–15 nm).^[^ ^13^ ^]^ Various nanoengineered surfaces and modification strategies have been explored to ensure that analyte binding occurs close to electrode surfaces, enabling sensitive detection of a broad spectrum of analytes, including protein and nucleic acid biomarkers. As research continues to address challenges related to sensitivity, selectivity, and real‐world applications, label‐free EIS biosensors are poised to play a vital role in the development of next‐generation biosensing technologies for widespread adoption in point‐of‐care (POC) and real‐time monitoring applications.

Thus, we focus our review on label‐free EIS biosensors where direct detection is achieved by monitoring interfacial capacitance changes and do not cover detection systems that utilize field‐effect transistors or nanopore‐based biosensors that work by sensing field‐effect modulation of current through a semiconducting channel or disruption in ionic current, respectively. We critically examine the fundamental principles, recent technological advancements, and ongoing challenges in the field of label‐free EIS biosensors. We also highlight their versatility, sensitivity, and suitability for direct detection, as well as their potential for transformative impact in clinical diagnostics, environmental monitoring, and beyond.

## 2 Fundamental Principles of EIS

EIS is a highly sensitive and versatile technique for probing complex electrochemical reactions occurring at an electrode/electrolyte interface.^[^ ^14^ ^]^ Unlike cyclic voltammetry or chronoamperometry which measure total current, EIS measures impedance over a range of frequencies allowing it to distinguish between various surface and bulk processes occurring within the detection system.^[^ ^10^, ^14^ ^]^ By analyzing the impedance response, detailed kinetic and mechanistic information, including rate constants, diffusion coefficients, and charge‐transfer resistances, reflecting these processes can be obtained.^[^ ^15^, ^16^ ^]^ In electrochemical biosensors, EIS is used to monitor shifts in interfacial properties that arise from specific biorecognition events.^[^ ^2^, ^10^ ^]^ While these measurements are simple to perform, analysis of the impedance response is complex and challenging. Moreover, diverse materials and surface chemistries contribute to intricate interfacial processes influencing the overall impedance response. Therefore, to truly appreciate the power of EIS, a comprehensive understanding of its fundamental principles, experimental limitations, and the intricacies of EIS data analysis is essential. This section aims to introduce the basic concepts of EIS, describe the electrochemical processes it can elucidate, and highlight the key factors impacting its applications in biosensor development.

### 2.1 Theory

Impedance is a foundational concept based on electric circuit theory, combining resistance, capacitance, and inductance to characterize how circuits respond to alternating current (AC). However, in the context of electrochemical biosensors, inductive effects are very rarely encountered. Therefore, this review will primarily focus on the resistive and capacitive effects typically observed in these systems.

When an electric circuit is perturbed either with a current (*I*) or voltage (*V*) signal, it generates a corresponding *V* or *I* response. In direct current (DC) theory, *I* through a simple resistor (energy dissipative element) is governed by Ohm's law (Equation (1)).

(1) $$I=\frac{V}{R}$$

where *R* or resistance represents the ability of a resistor to resist the flow of current. Similarly, *I* through the capacitor (energy storage element) is given by Equation (2)

(2) $$I=C\frac{dV}{dt}$$

where *C* represents the capacitance. The presence of time in Equation (2) highlights the frequency dependence of a capacitor. Unlike an ideal resistor, where *V* and *I* are always in phase and unaffected by frequency, capacitors introduce a phase shift between *V* and *I*. Consequently, their response cannot be fully described by Ohm's law and the concept of impedance becomes essential. In AC circuits, the impedance (*Z*) represents the total opposition (sum of resistive and capacitive opposition) that the circuit presents to the flow of AC and is defined by an equation analogous to Ohm's law (Equation (3)).

(3) $$Z=\frac{V}{I}$$

This distinction is crucial for understanding the electrochemical impedance characteristics of biosensors and fuel cells, which exhibit both resistive and capacitive components.

**Figure 1:**
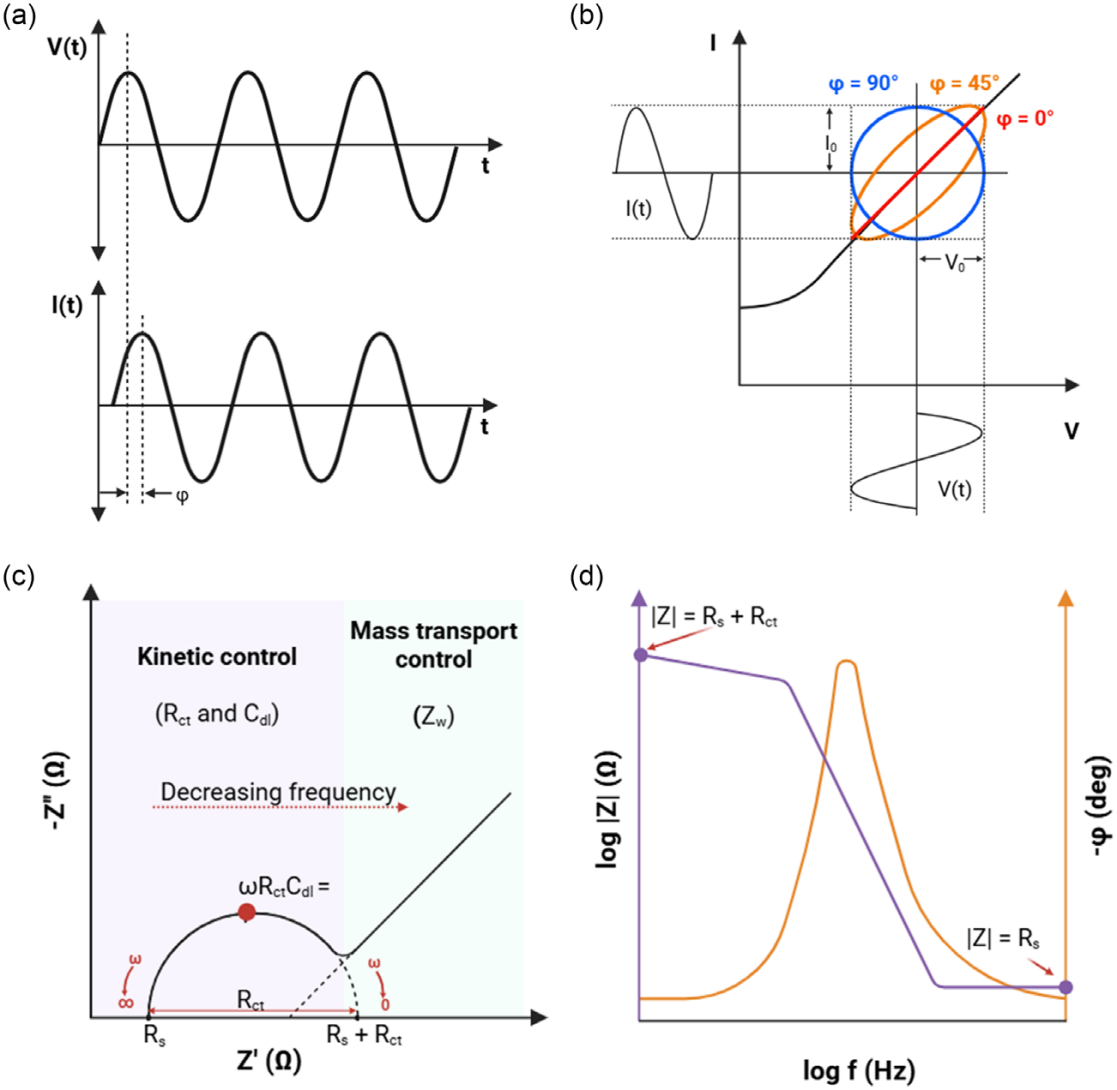
Schematic representation of typical EIS measurements for a linear (pseudolinear) system. a) Representation of applied potential and the corresponding current response in AC circuits. b) Lissajous plot showing relationship between potential and current response when *φ* = 0° or 45° or 90°. c) Nyquist plot of the impedance response across a range of frequencies, with *Z*′ on the x‐axis and *Z*″ on the y‐axis. d) Bode plot depicting the impedance magnitude and phase angle as a function of logarithmic frequency. Adapted with permission.^[^ ^14^ ^]^ copyright 2023, American Chemical Society (ACS).

In electrochemical cells, *Z* describes how the voltage applied across conductive electrodes relates to the movement of electrons in the aqueous solution. EIS is an electroanalytical technique that measures the system's response to a small sinusoidal perturbation, either potential (“potentiostatic EIS”) or current (“galvanostatic EIS”), thereby quantifying the system's impedimetric response.^[^ ^17^ ^]^ In potentiostatic EIS, when a small sinusoidal AC voltage perturbation *V*(*t*) is applied, the resulting current response *I*(*t*) for linear (or pseudolinear) systems will also be a sinusoidal wave oscillating at the same frequency but shifted in phase (*φ*) (**Figure** **1**a).

(4) $$V\left(t\right)=V_{0}\sin\left(\omega t\right)$$

(5) $$I\left(t\right)=I_{0}\sin\left(\omega t+\varphi \right)$$

(6) $$Z=\frac{V_{0}\sin\left(\omega t\right)}{I_{0}\sin\left(\omega t+\varphi \right)}=\;Z_{0}\frac{\sin\left(\omega t\right)}{\sin\left(\omega t+\varphi \right)}$$

where *V*_0_ and *I*_0_ represent the amplitude of *V*(*t*) and *I*(*t*), respectively, *ω* is the angular frequency, and *t* is the time. Based on Euler's relationship, *Z* (Equation (6)) when described in polar coordinates is a complex number possessing both magnitude (*Z*_0_) and phase (*φ*).

(7) $$Z=Z_{0}\exp\left(j\varphi \right)=\;Z_{0}\;(\cos\varphi +j\sin\varphi )$$

where *j* is the imaginary unit and *j* ^2^ = −1. In the Cartesian plane, *Z* is described as

(8) Z=Z′+jZ″

Thus, *Z* is a complex quantity consisting of a real part (resistance, *Z*′), which represents energy dissipation, and an imaginary part (reactive, *Z*″), which represents the circuit's ability to store and release energy.

### 2.2 Data Representation

The complex nature of impedance necessitates specific data representation methods, such as a Lissajous plot (time domain) or Nyquist and Bode plot (frequency domain), to fully capture the electrochemical system behavior (Figure 1). While modern EIS applications primarily use Nyquist and Bode representations, Lissajous plots remain valuable for the rapid assessment of impedance characteristics that may not be immediately apparent in these frequency domain plots.^[^ ^14^ ^]^ Lissajous plots offer visual representation of the instantaneous relationship between *V*(*t*) and *I*(*t*) at a given frequency on an XY plot reflecting the dominant electrical characteristics: a straight line (*φ* = 0°) or a circle (*φ* = 90°) indicates purely resistive or ideal capacitor behavior, respectively. However, in biosensors, an elliptical plot suggests a combination of resistive and capacitive components, indicating that complex binding events are occurring. Additionally, this plot provides key insights into both the linearity and time invariance (steady‐state property) of an electrochemical system during EIS measurement.^[^ ^18^ ^]^ For a linear system, the Lissajous plot exhibits central symmetry, typically forming a perfect ellipse. But electrochemical biosensors are not perfectly linear and instead demonstrate a pseudolinear behavior, and any deviation from elliptical shape indicates nonlinear behavior. But these plots are qualitative and require analyzing multiple single‐frequency plots to construct a complete impedance spectrum, and hence, they are not suited for comprehensive analysis.

On the other hand, Nyquist and Bode plots provide a concise summary of the impedance response across a range of frequencies, facilitating detailed, quantitative interpretation of EIS data and extraction of key system parameters. The Nyquist plot displays the overall impedance characteristics by plotting *Z′* on the x‐axis against *−Z″* on the y‐axis; each point represents *Z* at a specific frequency, and frequencies are represented in reverse order (i.e., high to low frequencies from left to right) (Figure 1c). This is the most frequently used convention to represent EIS data, and it is a common rule that these plots have equal‐scaled axes, especially for effective assessment of circularity that enables interpretation of various electrochemical processes. But this rule is often overlooked, which can obscure accurate analysis by diminishing the visibility of key features in the data. In contrast, a Bode plot shows EIS data as two separate graphs where magnitude and phase angle are plotted against logarithm of frequency (Figure 1d). This direct mapping of impedance and phase to frequency facilitates easier identification of transitions between different electrochemical processes, which can be more challenging to discern in Nyquist plots where frequency is not explicitly shown.

### 2.3 Transduction Mechanisms

The electrode configuration is a critical factor that significantly impacts the performance of electrochemical biosensors. The biosensor may be configured with two, three, or four electrodes depending on potential control requirements and experimental objectives. When AC potential is applied, the current flows between the working electrode (WE) and counter electrode (CE), while the potential between the WE and reference electrode (RE) is measured to characterize the sensor's impedimetric response. To ensure CE does not limit reactions at WE, the surface area of CE must be sufficiently larger (i.e., at least three times larger) than that of the WE. Modern instrumentation often includes a fourth lead, known as a working sense (S) in four‐electrode configuration, although in both two‐ and three‐electrode configurations the S lead is typically connected with the WE lead.

While the simpler configuration is a two‐electrode setup where the CE and RE are connected, the most commonly preferred configuration by far is the three‐electrode setup for accurate measurements of electrode processes. This is due to the potential drift that can occur at the WE in a two‐electrode configuration, caused by the inability to maintain constant potential at CE and RE, which can compromise the accuracy of measurements and negatively affect biosensor performance characteristics. As a result, the two‐electrode configuration is typically suited for applications focused on overall impedance characterization rather than detailed mechanistic studies. In contrast, three‐electrode systems enable precise control of potential at the WE, enabling accurate characterization of interfacial phenomenon, such as biomolecular interactions and electron transfer processes. This leads to more reliable, reproducible, and sensitive biosensors, making the three‐electrode configuration the standard for most biodetection applications. The four‐electrode configuration further enhances accuracy, especially in very low impedance systems. By using separate current‐carrying and potential‐sensing electrodes, this configuration enables characterization of bulk processes occurring within the electrolyte, e.g., measure electrocatalytic processes or ion transport at membrane–liquid or liquid–liquid junctions in solid‐state cells. Four‐electrode configurations are also valuable for tissue engineering applications, such as monitoring ion transport in cellular microenvironments or providing valuable mechanistic details during cell growth and differentiation in a nondestructive manner.^[^ ^19^ ^]^

When an electrode is immersed in an electrolyte, the charges redistribute at the electrode interface to establish an electrochemical equilibrium.^[^ ^20^ ^]^ In conventional EIS, a small amplitude AC sinusoidal voltage is applied over a range of frequencies, perturbing this equilibrium. This perturbation results in the movement of charge carriers (electrons in the electrodes and ions in the electrolyte), resulting in the formation of an EDL. These changes either impede or facilitate electron flow affecting parameters, such as conductivity, permittivity, and resistivity, which can be interpreted by EIS measurements to deduce the system's electrochemical behavior. The charge perturbation at the interface involves both energy dissipation through the movement of charged particles (electrons or ions) and energy storage via the accumulation or depletion of charges in the EDL or within intercalation materials that enable reversible ion storage and release.^[^ ^21^ ^]^ The energy dissipation process, which is often referred to as Faradaic, typically occurs in the presence of redox‐active molecules, while the energy storage process that is non‐Faradaic occurs in their absence. Selecting the most appropriate technique for biosensor development depends on several factors, including the desired transducer properties, electrochemical characteristics of interest, and the specific application. While the electrode materials and surface modification impact the sensitivity and specificity of biosensor, the choice of EIS method also plays a crucial role in optimizing biosensor performance.

#### 2.3.1 Faradaic EIS

Faradaic EIS is a labeled electrochemical technique which leverages redox‐active species to facilitate charge transfer at the electrode/electrolyte interface. The charge transfer is coupled with redox reactions occurring at the interface either due to the oxidation or reduction of redox‐active molecules present in the electrolyte or immobilized on the electrode surface. This process facilitates the movement of charged particles from one bulk phase to another, effectively coupling electronic to ionic processes and vice versa across the interface.^[^ ^21^ ^]^ When the potential is applied, the system responds with a current that is proportional to the redox reaction kinetics, mass transport, and diffusion, providing insights into the nature of interfacial processes and electrochemical behavior. The current generated by redox reactions obeys Faraday's law of electrolysis which states that the magnitude of current generated is proportional to the total charge through the system.^[^ ^22^ ^]^

(9) I=nFAj′

where *I* is the Faradaic current, *n* is the number of electrons, *F* is the Faraday constant, *A* is the surface area, and *j′* is the flux of electroactive species. Typically, EIS measurements employ an equimolar concentration of oxidized and reduced forms of a redox species to maintain equilibrium potential at the interface. This ensures that the open circuit potential is fixed at the formal potential (potential of an electrode or redox molecule under well‐defined experimental conditions), which is crucial for reliable and reproducible EIS measurements. Common redox probes include potassium ferrocyanide/ferricyanide ([Fe(CN)_6_]^4−^/[Fe(CN)_6_]^3−^), ruthenium hexamine ([Ru(NH_3_)_6_]^4+^/[Ru(NH_3_)_6_]^2+^), and others, chosen for their rapid electron transfer kinetics and chemical stability. Although voltammetric or amperometric techniques are most commonly used to characterize these reactions, EIS is particularly valuable for probing charge transfer kinetics, double‐layer capacitance, and diffusion processes. Unlike non‐Faradaic EIS, Faradaic EIS offers significantly higher sensitivity due to direct involvement of electron transfer reactions.^[^ ^23^ ^]^ This makes it a preferred method in biosensing applications that require very high sensitivity and specificity for detecting target analytes.

#### 2.3.2 Non‐Faradaic EIS

Non‐Faradaic EIS is a label‐free analytical method that focuses primarily on the properties of the EDL formed at the electrode/electrolyte interface to characterize electrochemical behavior. Non‐Faradaic EIS does not involve redox reactions and instead relies on charge perturbations resulting from the accumulation of ions or other chemical species at the electrode surface which results in the formation of compact, heterogeneous layers at the electrode/electrolyte interface that progressively leads to charge storage within the EDL. This involves charging/discharging of the EDL as well as adsorption and desorption of ions, all of which ultimately leads to changes in the interfacial properties, including dielectric constant, capacitance, and resistance that are directly reflected in the impedance characteristics. Consequently, these processes result in a transient current that decays over time as the system approaches equilibrium. The measured impedance mostly reflects EDL capacitance (*C*_dl_), which can be described using Equation (10).

(10) $$C_{\text{dl}}=\;\frac{\varepsilon_{0}\varepsilon_{r}A}{d}$$

where $\varepsilon_{0}$ and $\varepsilon_{r}$ are the permittivity of free space and solution (dielectric constant), respectively. Here, *A* represents the surface area of the electrode and *d* is the thickness of the EDL (i.e., distance between charged layers). *C*_dl_ obeys a power law of frequency in that *C*_dl_ decreases with an increase in AC frequency, which can be exploited to detect specific target analytes and enhance biosensor performance.^[^ ^24^ ^]^ Additionally, because non‐Faradaic EIS does not require additional DC bias typically used to drive redox reactions, the use of RE is not a must, and therefore, a two‐electrode configuration can be used. This further simplifies sensor design, making non‐Faradaic sensors highly amenable to miniaturization and suitable for portable and real‐time applications.

### 2.4 Equivalent Circuits

To extract quantitative mechanistic information about various electrochemical processes, the impedance spectra, mostly Nyquist plots, are fitted to appropriate equivalent electrical circuit models or more advanced mathematical frameworks.^[^ ^25^ ^]^ Typically, these circuits are composed of both lumped and distributed elements connected in series and/or parallel configurations depending on the electrode and surface modification characteristics.^[^ ^17^ ^]^ Lumped elements, which includes simple resistors and capacitors usually described by ordinary differential equations as mentioned in Section 2.1, are suitable for describing processes that are spatially uniform. However, complex electrochemical characteristics, such as charge transfer and diffusion, are distributed in space across the interface, and they can only be described using partial differential equations. Therefore, distributed elements like constant phase element (CPE) and Warburg element (*W*) are used to represent nonideal capacitance behavior and diffusion, respectively.^[^ ^25^ ^]^ Some of the most important circuit elements include the following. 1) Solution resistance (*R*_s_): This represents the resistance that the ions experience within the bulk electrolyte and contributes to overall internal resistance of the electrochemical system. *R*_s_ is influenced by ionic concentration and electrode geometry. Its value can be obtained from the high‐frequency intercept on the x‐axis of the Nyquist plot. 2) Charge transfer resistance (*R*_ct_): This represents the resistance that solvated ions experience at the interface when electron transfer takes place. In the Nyquist plot, *R*_ct_ is represented by the diameter of the semicircle. *R*_ct_ reflects the reaction kinetics, and lower *R*_ct_ represents faster reaction rates, while higher *R*_ct_ signifies slower reaction rates. 3) *C*_dl_/CPE: *C*_dl_ arises from the formation of EDL at the interface which resembles a parallel plate capacitor. However, *C*_dl_ is often replaced by CPE, which represents pseudocapacitance due to nonuniform charge distribution and heterogeneous surface properties. This is typically observed in biosensors, where the nature of biomolecular interactions in addition to surface roughness results in a nonideal capacitive behavior. 4) Warburg element (*W*): This is used to represent impedance due to diffusion of electroactive species to and from the electrode. For semi‐infinite diffusion, it typically appears as a straight line with 45° slope at low frequencies in the Nyquist plot. In case of hemispherical diffusion, a nonlinear resistance is included in parallel with *W* to represent the diffusion process at the micro/nanoelectrodes.^[^ ^26^ ^]^

**Figure 2:**
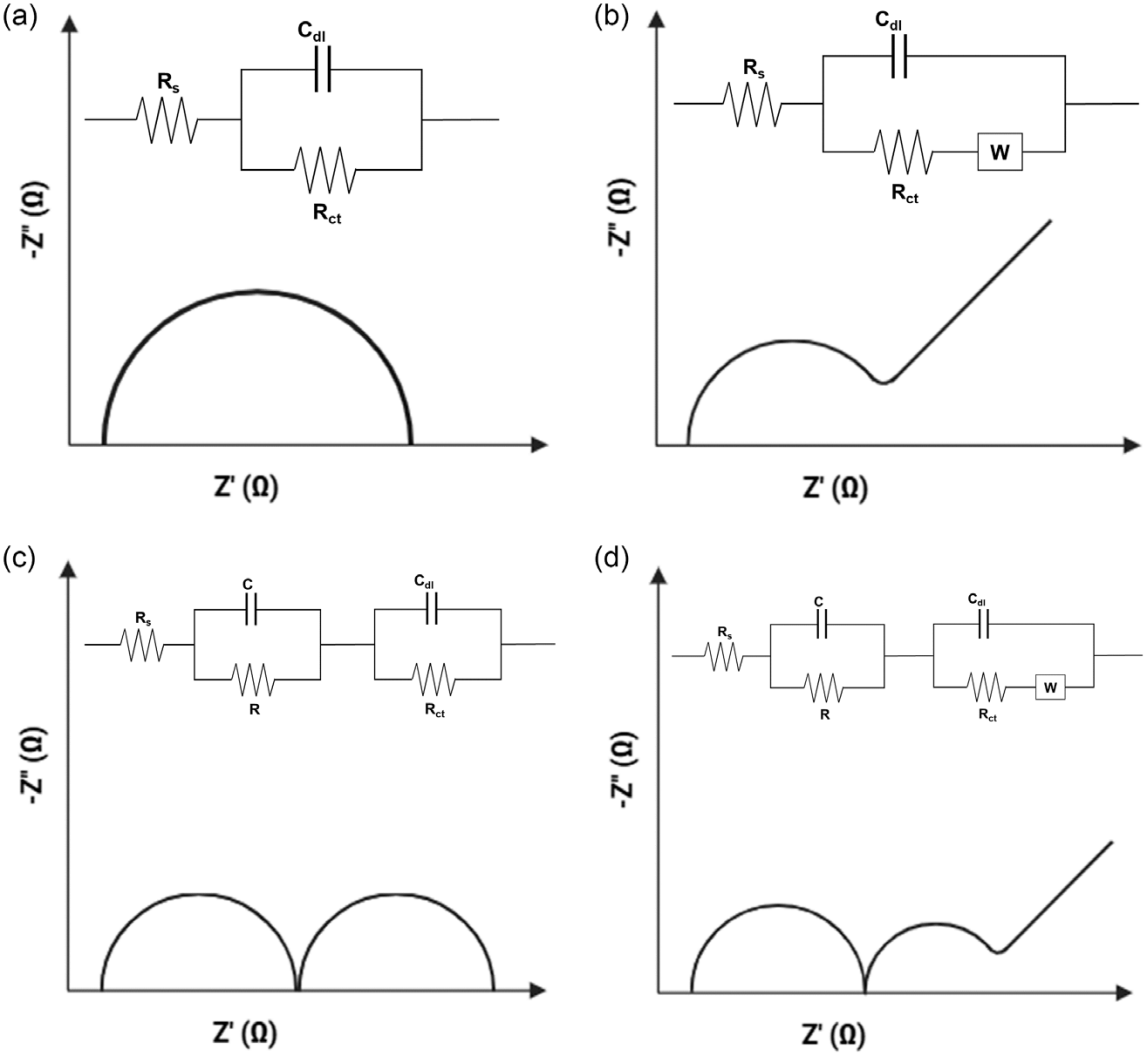
Comparison of Nyquist plots and their associated equivalent circuit models commonly observed in electrochemical biosensors. Reproduced with permission.^[^ ^27^ ^]^ Copyright 2014, Elsevier.

Different electrode surface modifications lead to distinct changes in these parameters, and a well‐chosen equivalent circuit is critical for meaningful interpretation and accurate prediction of electrochemical characteristics.^[^ ^27^ ^]^ For instance, the Nyquist plot for a bare gold electrode typically exhibits a semicircle whose diameter corresponds to *R*_ct_ and a low‐frequency region reflecting *W*. When the gold electrode surface is modified with an insulating or biomolecular layer, it hinders electron transfer leading to an increase in *R*_ct_.^[^ ^28^, ^29^ ^]^ In capacitive sensors, this modification also reduces *C*_dl_ as the biomolecular layer acts as a dielectric barrier limiting interfacial charge storage.^[^ ^30^ ^]^ Conversely, surface modification with conductive nanomaterials may reduce *R*_ct_ and enhance *C*_dl_.^[^ ^31^ ^]^ The interplay between these parameters ultimately influences biosensor sensitivity and its response characteristics. Typically, a parallel combination of *R*_ct_ and *C*_dl_/CPE is used to represent the semicircle typically observed in Nyquist plots (**Figure** **2**a). One of the most commonly used models in biosensors is Randle's circuit, which consists of *R*_s_ in series with parallel combination of *R*_ct_ and *W* with *C*_dl_/CPE (Figure 2b). Complex surface modifications or multilayered sensor architecture can present additional interfacial properties, where additional resistive and capacitive elements are included in the equivalent circuits (Figure 2c,d). For example, in living cell‐based systems, the dielectric properties of cell membrane and resistance between cells are represented as an additional capacitor (*C*) and resistor (*R*) in parallel with a basic Randle's circuit (Figure 2c,d). Any alterations in cellular processes, such as proliferation, shape, or attachment, can cause *C* and *R* to change, which is directly reflected as changes in the impedance response.^[^ ^19^ ^]^

### 2.5 Mechanistic Factors Impacting Biosensor Performance

The performance of EIS biosensors is mechanistically governed by a combination of physical, chemical, and biological factors that influence sensitivity, selectivity, accuracy, and reliability of impedance response. Specifically, the underlying mechanisms involve complex interfacial processes at the electrode/electrolyte interface modulated by both specific and nonspecific interactions. In complex biological or environmental samples, nonspecific interactions impede specific signal responses by causing impedance changes unrelated to target binding.^[^ ^32^ ^]^ Such interactions arise from the random adsorption of matrix components at defect or heterogeneous sites on the electrode surfaces which distort the EDL and introduce steric hindrances and alter overall interfacial conductivity, which manifest as a complex impedance spectra across a broad frequency range. These undesired effects typically lead to false positives, reduced sensitivity, and baseline drift.^[^ ^33^, ^34^ ^]^ On the other hand, specific binding events at the functionalized electrode surfaces involve localized, high‐affinity interactions, which form a uniform insulating layer altering *C*_dl_ and *R*_ct_ at specific frequencies enabling correlation to analyte concentration and binding kinetics.^[^ ^32^ ^]^ For example, in aptamer‐based biosensors, target binding induces conformational shifts which get reflected in the resulting impedance response.^[^ ^35^ ^]^ These changes are more pronounced at specific frequencies, where the sensor response shows the highest signal‐to‐noise ratio, and detection at the optimal frequency enhances sensitivity.

The nature of surface‐engineered electrode surfaces and immobilization of biorecognition elements influence the EDL characteristics at the electrode/electrolyte interfaces which dramatically affects the biosensor sensitivity and dynamic range. Additionally, the amplitude and frequency of the applied AC signals should be optimized to ensure detection of subtle impedance changes within the linear response range and maximize sensitivity while minimizing background noise and nonspecific effects.^[^ ^36^ ^]^ To mitigate nonspecific adsorption and improve signal reliability, electrodes are often coated with blocking such as bovine serum albumin (BSA), poly(ethylene) glycol (PEG), or zwitterionic polymers to create antifouling surfaces.^[^ ^37^ ^]^ Other mitigation strategies include employing engineered surface chemistries to enhance selective molecular recognition, differential or multifrequency measurements to isolate specific signals, and coupling EIS with other complementary techniques such as surface plasmon resonance for validation.^[^ ^38^ ^]^ From a mechanistic standpoint, reducing nonspecific adsorption through surface modification and selective molecular recognition events, combined with rigorous experimental control, is required to ensure that the impedance changes in label‐free EIS biosensors are predominantly derived from specific binding events. This approach is critical for reliable data acquisition and accurate interpretation of impedance spectra as well as ensure robust biosensor performance.

## 3 Material and Surface Modification Strategies

Label‐free EIS biosensors have seen significant progress through the combined use of advanced biorecognition elements and nanostructured materials. The synergistic combination of electrode materials and surface modification plays a vital role in determining the overall performance of these biosensors. Carbon, gold, silver, and metal oxides are commonly employed as electrode materials due to their excellent conductivity, ease of surface functionalization, and biocompatibility. Surface modification strategies are essential for immobilizing biorecognition elements on these electrode surfaces. Recent advances in surface engineering, such as the incorporation of nanomaterials and the development of nanostructured surfaces, have substantially improved sensor sensitivity by enhancing surface area, electron transfer, and biomolecule immobilization. Nanostructured surfaces typically not only reduce charge screening, demonstrating increased *C*_dl_ due to improved interfacial properties, but also introduce surface heterogeneity and roughness. Furthermore, nanostructured electrodes improve diffusion near the electrode surfaces enhancing sensitivity.^[^ ^20^ ^]^ Other advances, such as the use of nanocomposite films or polymer coatings, have improved the analytical performance of these biosensors by minimizing nonspecific binding and improving signal‐to‐noise ratio.^[^ ^7^, ^39^ ^]^

Fabrication methods such as drop‐casting, electrochemical deposition, and self‐assembly, combined with robust immobilization strategies (e.g., covalent bonding and self‐assembled monolayers (SAMs)) ensure stable and functional sensing interfaces.^[^ ^7^ ^]^ In parallel, micro‐ and nanofabrication techniques—such as microfluidics, nanolithography, and the development of microelectrode arrays—have supported the creation of compact multiplexed platforms.^[^ ^40^, ^41^ ^]^ Together, these advances reflect a cross‐disciplinary integration of materials science and engineering, enabling the development of highly sensitive, selective, and scalable biosensors for real‐time detection in complex environments, expanding the scope and impact of EIS‐based label‐free biosensing technologies.

### 3.1 Biorecognition Elements

A diverse array of biorecognition elements has been developed for the selective detection of target analytes in label‐free EIS biosensors. These include naturally derived or engineered components such as antibodies, nucleic acids, enzymes, coenzymes, whole cells, and affinity ligands, as well as synthetic constructs like peptides, aptamers, and molecularly imprinted polymers (MIPs).^[^ ^42^, ^43^ ^]^ Specifically, in the context of ion detection, specialized elements, such as double‐stranded nucleic acids, DNAzymes, and ionophores, are commonly employed. Central to effective biosensing is the principle of molecular recognition, which underpins the specificity and sensitivity of the biosensor. While many recognition elements were originally sourced from biological systems, advances in synthetic biology and materials science have enabled laboratory‐based creation of highly selective alternatives. Among these, aptamers and nucleic acid ligands developed through the Systematic Evolution of Ligands by Exponential Enrichment (SELEX) process are especially notable for their ability to fold into complex 3D shapes, allowing them to bind target molecules with high affinity and precision.^[^ ^44^ ^]^ Immobilization of biorecognition elements and subsequent analyte binding can form an insulating or semi‐insulating layer at the electrode/electrolyte interfaces altering electron transfer and dielectric properties leading to measurable changes in *R*_ct_ and *C*_dl_ or CPE, which can be correlated to analyte concentration.

#### 3.1.1 Antibody

Antibodies, owing to their high specificity and affinity, are widely employed as biorecognition elements in label‐free EIS biosensors. These biosensors typically rely on the immobilization of antibodies on conductive electrode surfaces, commonly gold or carbon, via methods such as EDC/NHS coupling, SAMs, or passive adsorption. The method of antibody immobilization significantly influences the sensitivity and specificity of EIS biosensors.^[^ ^45^ ^]^ Covalent immobilization using EDC/NHS chemistry is widely employed due to its stability.^[^ ^46^ ^]^ However, this approach can result in random antibody orientation, potentially reducing antigen binding efficiency.

**Figure 3:**
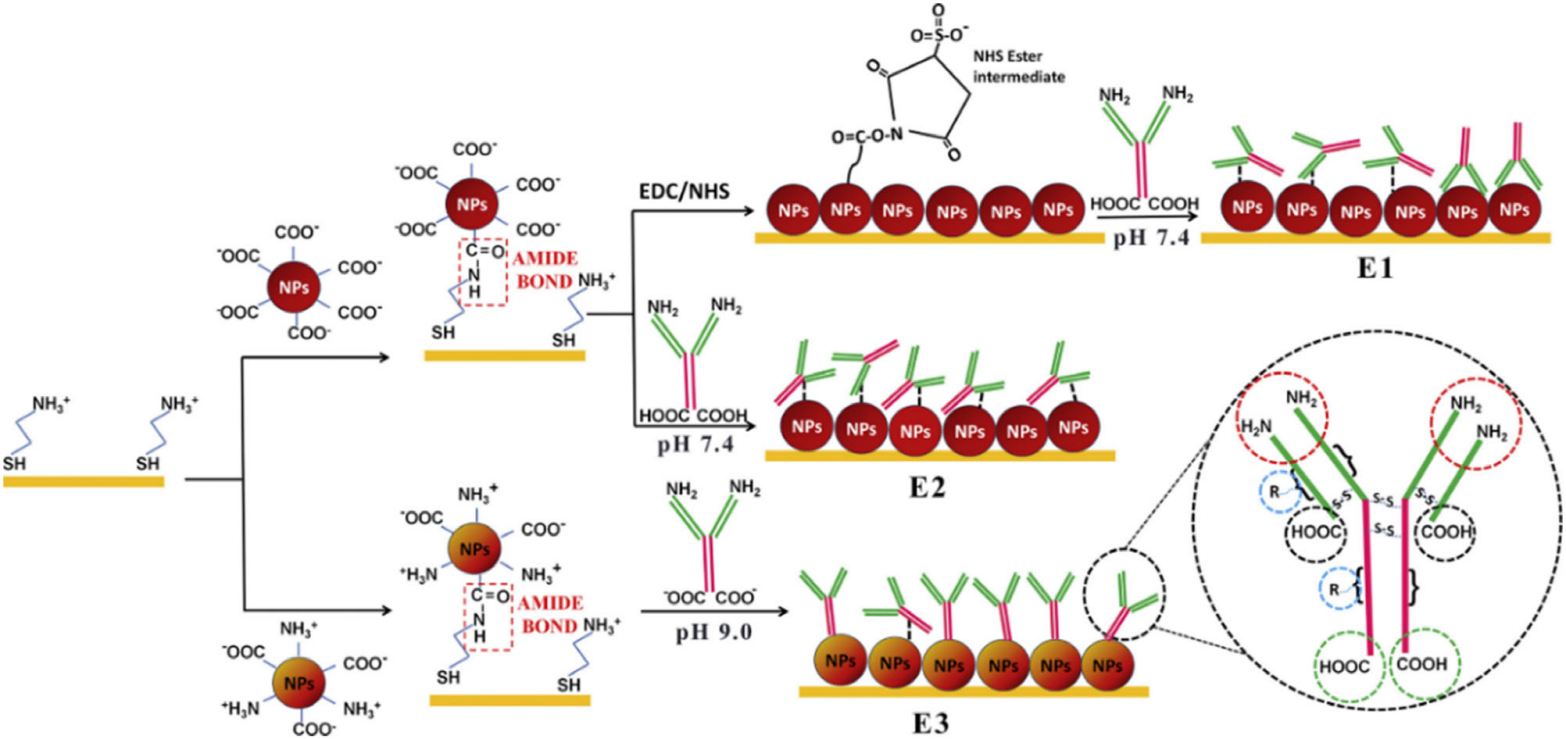
Layer‐by‐layer modification of screen‐printed electrode surface for (1) E1‐cit–AuNP–EDC–NHS–AntiCA125, (2) E2‐cit–AuNP–AntiCA125, and (3) E3–Asn–AuNP–AntiCA125 immunosensor fabrication. Reproduced with permission.^[^ ^49^ ^]^ Copyright 2016, Elsevier.

Immobilized antibodies create a functionalized interface capable of selectively capturing target analytes. Upon antigen binding, subtle physicochemical changes occur at the electrode–electrolyte interface, which induces measurable changes in the interfacial properties, such as *R*_ct_ and *C*_dl_, which are detectable by EIS. This sensitivity makes EIS suitable for detecting binding of small molecules, such as norfluoxetine and BDE‐47.^[^ ^47^, ^48^ ^]^ But, antibodies are typically 10–15 nm in length, which is significantly larger than the Debye length under physiological conditions. Therefore, antigen binding may occur far beyond the Debye length where the electric potential exponentially decays from the electrode surface into the electrolytic solution. As a result, changes to *C*_dl_ may be screened by mobile ions in the electrolyte, leading to signal attenuation and reduced sensor sensitivity.^[^ ^12^ ^]^ One of the strategies employed to optimize antibody orientation and ensure that antigen binding occurs close to the electrode surface is site‐specific immobilization. For instance, immobilization using L‐asparagine‐capped gold nanoparticles (AuNPs) has demonstrated improved antibody orientation and increased sensitivity (**Figure** **3**).^[^ ^46^, ^49^ ^]^

Another approach involves immobilizing biotinylated antibodies onto streptavidin that is covalently attached to gold nanostructured screen‐printed carbon electrodes using 3‐mercaptopropionic acid.^[^ ^50^ ^]^ This strategy allowed for controlled and uniform orientation of biotinylated anti‐N‐protein antibodies enabling sensitive detection of the SARS‐CoV‐2 nucleocapsid protein.^[^ ^50^ ^]^ The label‐free nature of this approach enables direct, real‐time analysis and reduces assay complexity and cost. Sensor performance is highly dependent on the orientation and functional integrity of immobilized antibodies, necessitating careful surface engineering and the use of blocking agents such as BSA to minimize nonspecific binding.^[^ ^48^ ^]^ Other strategies for achieving directional antibody immobilization involve leveraging the specific affinity of protein A and protein G for the Fc region of antibodies.^[^ ^51^, ^52^ ^]^

Antibody‐based impedance biosensors demonstrate excellent selectivity in complex sample matrices, ranging from biological fluids to environmental and food‐related samples, making them particularly suited for applications where biological sample matrix effects can hinder conventional detection methods.^[^ ^53^ ^]^ Reversible antibody–antigen binding also allows for sensor regeneration through mild chemical treatments, enhancing operational longevity and cost‐effectiveness.^[^ ^54^, ^55^ ^]^ Nonetheless, challenges persist, particularly in ensuring reproducible antibody immobilization and maintaining bioactivity under operational conditions. Advances in recombinant antibody technologies, such as fragment engineering and site‐specific conjugation, along with innovations in nanostructured electrode materials, are progressively addressing these limitations.^[^ ^46^, ^56^ ^]^ Antigen‐binding fragments (Fab), single‐chain variable fragments (scFV), and nanobodies (NB) maintain high specificity and affinity for antigen targets, while the smaller size of these biorecognition elements enables them to overcome the Debye length limitation, improving signal resolution and sensitivity. For example, a nanobody‐based label‐free biosensor has been developed to detect the SARS‐CoV‐2 spike protein^[^ ^57^ ^]^ and a scFV‐based, label‐free EIS biosensor has been described for monitoring porcine circovirus type 2 (PCV2) in swine populations.^[^ ^58^ ^]^ With increasing PCV2 capsid concentrations, the impedance response (*R*_ct_) from scFVs immobilized on gold electrodes increased, achieving a detection limit of 114 nM.

Another promising alternative to antibodies are engineered protein scaffolds called affimers. These engineered molecules are highly specific and demonstrate high affinity for analytes and stability over a broad pH range, making them robust and reliable biorecognition elements for biosensor development. A label‐free affimer‐based sensor demonstrated high sensitivity for Her4 protein biomarker detection with excellent selectivity and a low limit of detection (LOD, 1 pM) in undiluted serum, which contains high concentrations of mobile ions.^[^ ^59^ ^]^ While these demonstrations open up a new avenue for developing label‐free EIS biosensors and hold promise for creating robust platforms for the sensitive and selective detection of biomolecules across a wide range of analytical contexts, continued research is needed to address critical challenges and ensure adoption in real‐world applications.

#### 3.1.2 Nucleic Acids and Aptamers

Nucleic acids, particularly aptamers, have become increasingly prominent as biorecognition elements in the development of label‐free EIS biosensors. These biosensors leverage intrinsic electrical properties and unique molecular recognition characteristics of nucleic acids, such as DNA, peptide nucleic acids (PNAs), and aptamers. PNAs and aptamers are short oligonucleotide sequences, often around 20–100 nucleotides, which when used as biorecognition elements allow hybridization events to occur in close proximity to electrode surfaces. PNAs are synthetic DNA analogs with neutral charge and, therefore, minimize electrostatic repulsion at the electrode/electrolyte interface. Upon hybridization of negatively charged DNA, significant increases in *R*_ct_ and *C*_dl_ are induced, which has been leveraged for biomolecular detection.^[^ ^60^ ^]^

Aptamers are short, single‐stranded DNA or RNA sequences selected through the SELEX process, which enables the identification of ligands with high affinity and specificity for a given target.^[^ ^61^, ^62^ ^]^ One of the key advantages of aptamers lies in their ability to fold into well‐defined 3D structures. This conformational flexibility allows for precise molecular recognition and is particularly well suited to EIS, where target binding often induces structural rearrangements that alter interfacial properties such as *R*_ct_, dielectric constant, and *C*_dl_.^[^ ^63^ ^]^ These alterations can be sensitively captured by EIS without the need for secondary labels, making the overall assay simpler, faster, and more cost‐effective.^[^ ^64^ ^]^

**Figure 4:**
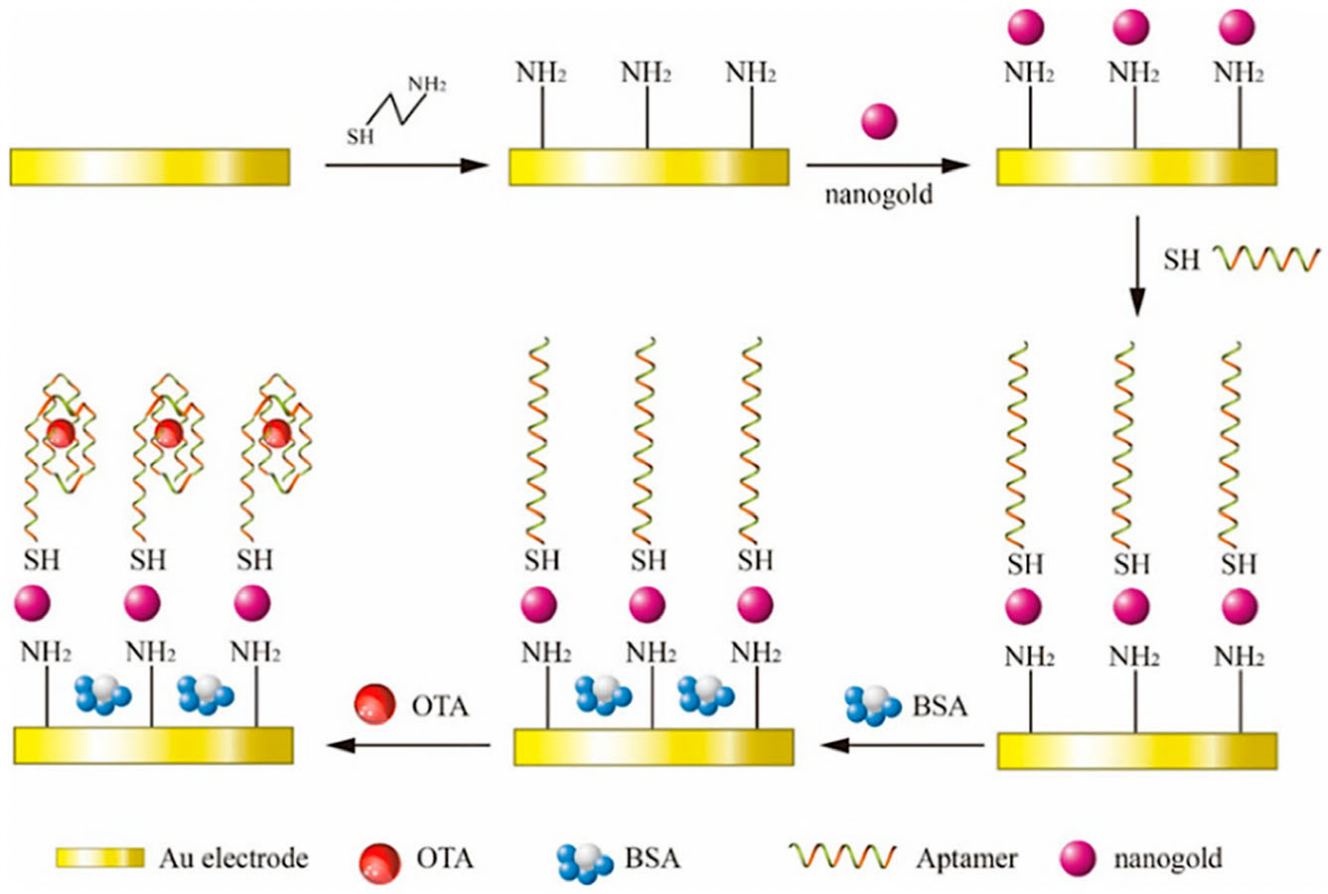
Schematic representation of the fabrication and principle of the label‐free impedance aptasensor for detection of Ochratoxin A in agricultural grape samples. Reproduced with permission.^[^ ^68^ ^]^ Copyright 2020, Elsevier Ltd.

Aptamers can be immobilized onto various electrode surfaces, including gold, glassy carbon, and nanostructured substrates, using well‐established surface chemistries such as thiol–gold self‐assembly, carbodiimide coupling, or biotin–streptavidin interactions.^[^ ^65^ ^]^ Their ability to undergo conformational changes upon target binding makes them particularly responsive to low‐molecular‐weight analytes, where signal amplification is inherently challenging.^[^ ^66^, ^67^ ^]^ Additionally, the terminal groups of aptamers can be modified with amino or thiol groups to enable direct immobilization on electrode surfaces. For example, one study compared the stability between amino‐ and thiol‐modified aptamers for ochratoxin A detection on gold electrodes by subjecting them to HCl treatment.^[^ ^68^ ^]^ Their results showed a 46% reduction in *R*_ct_ for amino‐modified aptasensor compared to only a 9% reduction for the thiol‐modified aptasensor (**Figure** **4**). While this study highlights superior stability and reusability of thiol‐modified aptasensors for up to 4 days, achieving long‐term stability (weeks to months) for commercial applications still remains a significant challenge. Another study reported the development of a highly sensitive aptasensor based on thiol‐terminated DNA aptamers for detecting human epidermal growth factor receptor 2 (HER2), a biomarker associated with breast cancer.^[^ ^29^ ^]^ The sensor demonstrated logarithmic detection of HER2 over a wide dynamic range in undiluted serum, highlighting the potential of aptamer‐based sensors for clinical diagnostics. Compared to antibodies, aptamers offer several practical advantages: they are produced synthetically, allowing for better reproducibility across batches, and they can be readily modified at specific sites to enhance stability, binding efficiency, or sensor integration.^[^ ^69^, ^70^ ^]^ Their superior chemical and thermal stability also supports broader application in field‐deployable and real‐time sensing formats.

Importantly, aptamer‐based sensors also can often be regenerated more easily than antibody‐based systems. Because aptamer‐target complexes frequently dissociate under mild changes in pH, ionic strength, or temperature, the same sensor surface can be reused multiple times without loss of function, an important advantage for operational sustainability and cost reduction.^[^ ^71^ ^]^ In addition, incorporating nanostructured materials such as carbon nanotubes (CNTs), graphene, and metallic nanoparticles has further enhanced the sensitivity and signal‐to‐noise ratio of aptamer‐based impedance biosensors by increasing effective surface area and improving probe orientation.^[^ ^68^, ^70^, ^72^ ^]^

#### 3.1.3 Enzymes

Enzymes and DNAzymes are increasingly being employed as biorecognition elements in label‐free EIS biosensors. This approach capitalizes on the inherent specificity and catalytic efficiency of enzymes to enable sensitive and selective analyte detection without the need for external labeling. Enzymes, such as acetylcholinesterase (AChE), have been particularly useful for detecting neurotransmitters. For example, an AChE biosensor was developed by covalently immobilizing AChE onto gold microelectrodes using dithiobis(succinimidyl propionate) (DSP).^[^ ^72^ ^]^ The resulting sensor exhibited a robust linear response to acetylcholine in the 5.5–550 μM range, demonstrating both high specificity and sensitivity in complex matrices including rat brain tissue and whole blood. The label‐free format not only simplified the assay workflow but also enhanced the sensor's suitability for real‐time applications.

DNAzymes—synthetic, catalytically active DNA molecules—have emerged as versatile alternatives due to their ability to undergo target‐induced cleavage reactions, as well as their structural adaptability and synthetic tunability. These enzymatic reactions produce pronounced changes in surface impedance, enabling highly sensitive detection. For instance, an electrochemical impedance biosensor for mercury ions (Hg^2+^) was developed that combines DNAzyme‐assisted signal recycling with a hybridization chain reaction.^[^ ^73^ ^]^ The system incorporated a DNA hydrogel layer that impeded electron transfer upon target binding, achieving an impressive detection limit of 0.042 pM. Such strategies demonstrate the powerful amplification potential of DNAzymes in impedance sensing. Further innovation has come from integrating DNAzymes with nanomaterials, as demonstrated by the development of a hybrid biosensor in which DNAzymes were immobilized on platinum nanoparticles for the detection of heavy metals such as Pb^2+^, Cd^2+^, and Cr^3+^.^[^ ^74^ ^]^ The cleavage of DNAzyme substrates by target ions disrupted conductive networks between the nanoparticles, leading to measurable increases in electrochemical impedance. This platform achieved detection limits as low as 0.8 nM for Pb^2+^, underscoring the utility of DNAzyme‐based biosensors in environmental monitoring.^[^ ^75^ ^]^ Together, enzyme‐ and DNAzyme‐based biosensors represent a promising avenue for label‐free, high‐sensitivity detection of biomolecules.

#### 3.1.4 Cell‐Based

Whole living cells, including both eukaryotic somatic cells and bacterial cells, are increasingly being used as biorecognition elements in label‐free EIS biosensors, where they serve as dynamic detection elements capable of translating biomolecule‐induced physiological changes into quantifiable impedance signals. In mammalian systems, hepatic cell lines such as HepG2, have been employed in microfluidic impedance setups to evaluate the real‐time toxicity of environmental chemicals and drug candidates. When cultured as monolayers or spheroids on interdigitated microelectrodes, these cells exhibit impedance changes in response to various compounds, including pharmaceuticals and azo dyes. These responses are often due to disruption in membrane integrity or tight junctions, or changes in metabolic activity, which alters the dielectric properties at the electrode–cell interface. EIS captures these alterations without the need for external labels, offering a continuous and noninvasive method to monitor cellular health and function.^[^ ^76^ ^]^ Importantly, these systems are sensitive enough to detect subtle, dose‐dependent cellular responses, even at sublethal concentrations, making them valuable for early‐stage screening of cytotoxicity, with detection limits in the low micromolar to nanomolar range.

Bacterial cells also have been leveraged for label‐free impedance biosensing, particularly for antibiotic detection. For instance, outer membrane vesicles (OMVs) derived from enterohemorrhagic *Escherichia coli* (*E. coli*) have been reconstituted onto conducting polymer substrates to create a responsive interface for monitoring antibiotic–membrane interactions. Binding of small‐molecule antibiotics, such as polymyxin B, bacitracin, and meropenem, to lipid and protein components of the membrane induces changes in interfacial impedance, particularly *R*_ct_, enabling detection in the femtomolar to nanomolar concentration range.^[^ ^77^ ^]^ In another example, intact *Bacillus subtilis* cells have been incorporated into biosensor platforms where the bacteria's native *σ* ^M^‐mediated stress‐response pathways act as transducers. Upon exposure to cell envelope‐active antibiotics, these systems exhibit measurable impedance shifts linked to cell wall perturbation, allowing selective detection of compounds, such as polymyxin B, within a 0.125–12 μg mL^−1^ range.^[^ ^78^ ^]^ Altogether, these approaches leverage the intrinsic complexity of cellular signaling and membrane architecture to achieve sensitive, label‐free detection, offering a physiologically relevant toolset for applications in toxicology, pharmacology, and antimicrobial screening.

### 3.2 Nanomaterials/Nanostructured Electrodes

The incorporation of nanomaterials and nanostructured surface chemistries into electrode design has significantly enhanced the performance and sensitivity of label‐free EIS biosensors for biomolecule detection. Materials such as CNTs, graphene and its derivatives, AuNPs, metal oxides, and various hybrid nanostructures offer numerous advantages, including increased surface area, improved electron transfer kinetics, and efficient immobilization of biorecognition elements.^[^ ^56^, ^79^, ^80^ ^]^ These properties collectively contribute to improved signal transduction and lower detection limits. For example, nanostructures like gold nanorods integrated with multiwalled CNTs (MWCNTs), gold nanoclusters, graphene nanoribbons, and graphene oxide–gold nanocomposites have been successfully employed to enhance the electrochemical detection of amino acids.^[^ ^81^ ^]^ More complex architectures, including caterpillar‐like MnO_2_/carbon nanocomposites and GaN nanowires, also have been used for general amino acid analysis,^[^ ^82^ ^]^ while platforms such as MWCNT‐bridged mesocellular graphene foam and silver‐based nanostructures have shown excellent sensitivity in tryptophan detection.^[^ ^83^, ^84^ ^]^ Graphene oxide nanoribbons and C_3_N_4_ nanosheets also have been used to sense tyrosine with high specificity, underscoring the adaptability of nanomaterial‐based platforms across a wide range of analytes.^[^ ^85^, ^86^ ^]^

The fabrication of these advanced sensing interfaces typically combines top–down and bottom–up approaches to optimize both surface morphology and functionalization. Techniques such as drop‐casting, electrochemical deposition, and self‐assembly are widely used to integrate nanomaterials onto electrode surfaces, improving their conductivity and electrochemical reactivity.^[^ ^87^ ^]^ In parallel, biorecognition elements are immobilized using covalent binding, physical adsorption, or entrapment strategies, often aided by SAMs to ensure consistent orientation and stable attachment. The use of microfabrication and nanofabrication methods, including the development of microelectrode arrays, integration with microfluidic systems, nanolithography, and electrospinning, has enabled the creation of compact, high‐throughput, and multiplexed biosensor platforms.^[^ ^40^, ^41^ ^]^ Additionally, molecular imprinting has emerged as a powerful tool for designing synthetic recognition sites tailored to specific analytes, offering a complementary approach to biological receptors. Together, these strategies represent a synergistic fusion of material science and sensor engineering, driving ongoing innovation in electrochemical biosensing and opening new possibilities for the sensitive, selective, and real‐time detection of biomolecules in complex environments.

#### 3.2.1 Polymers and Nanocomposites

**Figure 5:**
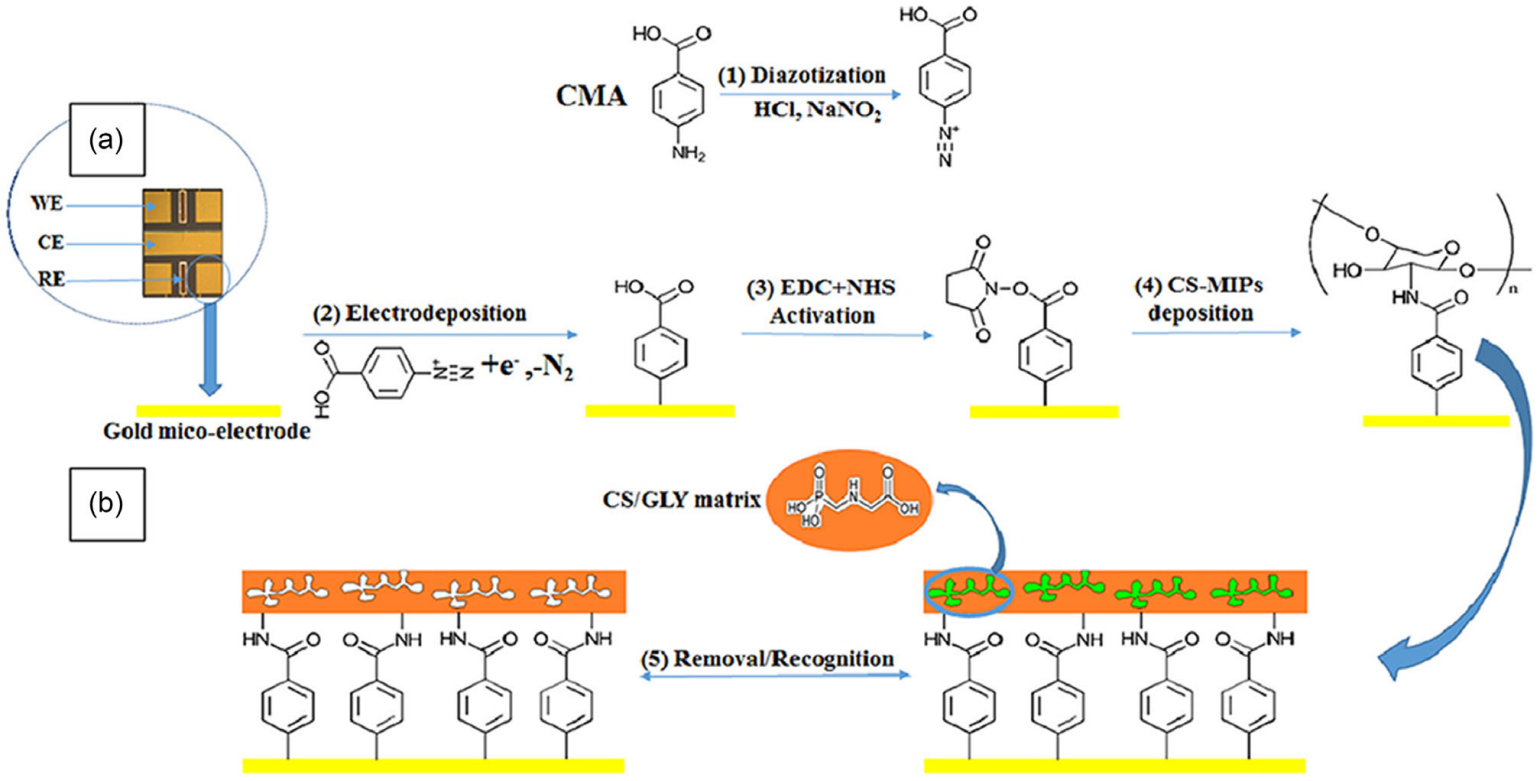
a) Transducer holding an array of four bare‐gold working microelectrodes, one counter microelectrode, and two Ag/AgCl reference microelectrodes. b) Preparation of chitosan‐MIPs/4‐aminophenylacetic acid/Au and its recognition for glyphosate. Reproduced with permission.^[^ ^91^ ^]^ Copyright 2021, Frontiers Media S.A.

Polymers and nanocomposites have become integral to the fabrication of nanostructured electrodes in label‐free EIS biosensors for small molecule detection. Their utility stems from tunable chemical functionalities, high surface area, and enhanced electrical conductivity, which together support efficient signal transduction and selective target recognition. One widely adopted strategy involves MIPs—synthetic materials that form molecular recognition sites complementary in shape, size, and functionality to specific analytes. For example, a polydopamine‐based MIP sensor was developed by immobilizing a thiolated aptamer‐PSA complex on gold electrodes.^[^ ^88^ ^]^ The sensor exhibits high *R*_ct_ that is consistent with the electrical characteristics of the MIP biosensor construct, which resulted in a threefold increase in sensitivity for PSA detection compared to their previous study, which reported a similar aptasensor without MIP.^[^ ^89^ ^]^ Another MIP sensor was fabricated by electropolymerizing a bioinspired polymethyldopa (PMD) film on a pencil graphite electrode in the presence of the antiviral drug sofosbuvir.^[^ ^90^ ^]^ After template extraction, the MIP exhibited highly selective recognition of this drug, achieving a detection limit of 31 fM through impedance measurements. Similarly, a MIP was constructed on gold microelectrodes by grafting chitosan onto 4‐aminophenylacetic acid‐modified surfaces.^[^ ^91^ ^]^ This platform enabled selective detection of the herbicide glyphosate, with a dynamic range from 30 fg mL^−1^ to 50 ng mL^−1^ and a detection limit as low as 1 fg mL^−1^ (**Figure** **5**). Another example involved the use of a bis(2,2′‐bithien‐5‐yl)methane‐based electropolymerized film to detect carnosine—a biologically relevant dipeptide—at concentrations as low as 20 μM in a flow‐system setup, demonstrating the adaptability of MIP designs for varied molecular targets.^[^ ^92^ ^]^

Beyond MIPs, polymer‐based nanocomposites—particularly those combining conductive polymers like polypyrrole (PPy) or polyaniline (PANI) with nanostructured materials, such as CNTs or metal oxides (e.g., ZnO)—offer additional performance enhancements. These hybrids improve electron transfer kinetics, support the immobilization of bioreceptors, and provide antifouling capabilities. In one study, a chitosan–ZnO/PANI nanocomposite‐modified electrode was developed for the selective detection of dopamine.^[^ ^93^ ^]^ The composite architecture enabled separation from interfering species and yielded a detection limit of 0.21 μM. Another study highlighted the synergistic effect of combining CNTs with PPy, facilitating fast charge transfer and high electrochemical sensitivity.^[^ ^94^ ^]^ Together, these approaches demonstrate how the combination of molecular recognition elements (like MIPs) with highly conductive polymer–nanomaterial scaffolds can translate subtle binding events into amplified impedance responses. This synergy continues to advance the field of label‐free biosensors, supporting the development of sensitive, real‐time platforms for varying clinical and field applications.

#### 3.2.2 Semiconductors

Semiconducting materials, particularly metal oxides and layered dichalcogenides, have emerged as valuable components in the design of nanostructured electrodes for label‐free EIS biosensors. Among these, zinc oxide (ZnO) stands out due to its high electron mobility and the ability to form single, crystalline, 1D nanostructures.^[^ ^95^ ^]^ Its unique physical characteristics enable the creation of diverse nanoarchitectures, including nanorods and nanowires, which offer an exceptionally large surface‐to‐volume ratio as well as unique interfacial properties that can be leveraged for label‐free biosensing.^[^ ^20^ ^]^ For example, a biosensor was created by growing nanorods directly on silver electrodes and functionalizing the surface with anti‐17β‐estradiol antibodies via silane chemistry.^[^ ^96^ ^]^ This configuration enabled highly sensitive, label‐free detection of the endocrine‐disrupting compound 17β‐estradiol, achieving an LOD as low as 0.1 pg mL^−1^ and demonstrating a robust, concentration‐dependent impedance response. Similarly, other research groups have demonstrated the use of ZnO thin films deposited on various substrate materials for sweat diagnostics^[^ ^97^ ^]^ and the detection of pathogens in water.^[^ ^98^ ^]^ However, the long‐term stability of ZnO biosensors is challenged by environmental stressors like light, humidity, and temperature, which can degrade sensor performance leading to decreased sensitivity and accuracy.

Titanium dioxide (TiO_2_) nanotube arrays (TNAs) offer another compelling semiconductor platform. While typically employed in photoelectrochemical contexts, they have also shown strong potential in label‐free impedance sensing. For example, cobalt‐functionalized TNAs were used to detect the receptor‐binding domain of the SARS‐CoV‐2 spike protein via impedance spectroscopy, achieving sub‐nanomolar sensitivity (≈0.7 nM) without the need for redox labels.^[^ ^99^ ^]^ This system not only leveraged the high surface area and stability of TNAs but also benefited from TiO_2_'s self‐cleaning photocatalytic properties, underlining the versatility of semiconductor‐based biosensors.

Recent developments have also focused on tin dioxide (SnO_2_), particularly in nanoporous and 1D nanostructured forms. For instance, a sensor was created by electrodepositing SnO_2_ films decorated with Cu_2_O nanoparticles for the detection of creatinine, a clinically relevant small molecule biomarker.^[^ ^100^ ^]^ This sensor achieved a detection limit of 2 nM and exhibited strong selectivity against common interferents. This selectivity was attributed to the synergy between SnO_2_'s adjustable bandgap and Cu_2_O's electrocatalytic behavior, which enhanced charge transfer kinetics.

In addition to metal oxides, 2D semiconductors such as molybdenum disulfide (MoS_2_) have been explored for their high surface area, electrical conductivity, and ease of functionalization. For example, a label‐free EIS‐based aptasensor was developed for thrombin detection based on chemically exfoliated monolayers of MoS_2_ nanosheets, which were dispersed in solution and drop‐casted onto Pt electrodes.^[^ ^101^ ^]^ While this sensor achieved an LOD of 53 pM for thrombin in 1% human serum, the study did not address the stability and shelf life of the MoS_2_‐aptamer interface. This is a serious concern as MoS_2_ is susceptible to oxidation and degradation, which may affect sensor reproducibility and longevity. While much of the work on MoS_2_ has centered on nucleic acid biosensing, its physicochemical properties suggest strong potential for other biomolecule targets (e.g., as neurotransmitters and metabolites) as well. Collectively, these examples underscore the critical role that semiconductors play in advancing the sensitivity, selectivity, and robustness of label‐free EIS biosensors across biomedical, environmental, and industrial applications.

#### 3.2.3 Metal‐Organic Frameworks

Metal‐organic frameworks (MOFs) have also garnered significant attention as promising materials for the fabrication of nanostructured electrodes in label‐free EIS biosensors, particularly for the detection of biomolecules. Their unique combination of highly tunable porosity, large surface area, and the presence of redox‐active sites allows for the development of sensitive and selective sensing platforms. These structural advantages make MOFs particularly effective as scaffolds for the immobilization of biomolecules, offering enhanced electron transfer properties and increased binding capacity. Notably, 2D MOF nanosheets have demonstrated particular utility due to their planar architecture, which exposes more active sites for biomolecular interactions, including π–π stacking, hydrogen bonding, and electrostatic forces. This enables the stable attachment of biorecognition elements, such as enzymes or aptamers, without requiring complex chemical modification steps. For example, a zirconium‐based 521‐MOF was recently employed to immobilize aptamers targeting Mucin‐1, a cancer‐associated glycoprotein.^[^ ^102^ ^]^ The resulting biosensor achieved a significantly low LOD of 0.12 pg mL^−1^ using EIS, underscoring the potential of MOF‐based electrodes for highly sensitive, label‐free analysis.^[^ ^102^, ^103^ ^]^ When applied to biomolecule detection, the performance of MOF‐based biosensors can be significantly enhanced by combining MOFs with conductive materials such as AuNPs, CNTs, or metal oxide nanoparticles. These hybrid architectures facilitate more efficient electron transfer across the electrode interface while preserving the high surface area and molecular selectivity of the MOF structure. For example, a zirconium‐based MOF/CNT composite platform was developed for label‐free electrochemical detection of CA‐125, a cancer biomarker.^[^ ^104^ ^]^ In this system, antibody binding to the immobilized target protein obstructed the diffusion of the ferrocyanide redox probe, resulting in measurable changes in *R*_ct_, as captured by impedance spectroscopy. The synergistic combination of MOF porosity with the conductive CNT network allowed for sensitive, label‐free quantification of the analyte. Similarly, copper‐based MOFs (Cu‐MOFs) uniformly decorated with AuNPs were developed for detection of methyl jasmonate, a plant hormone with roles in pest defense and developmental growth.^[^ ^105^ ^]^ In this case, the redox‐active Cu^2+^ centers in the MOF framework served as inherent signal transducers, while the AuNPs enhanced electron mobility, yielding distinct impedance shifts upon small molecule binding. These examples illustrate how coupling MOFs with conductive nanomaterials offers a promising route to engineer biosensors that combine molecular recognition with robust electrochemical readouts, particularly important for detecting low‐abundance biomolecules in food safety and environmental applications.

**Figure 6:**
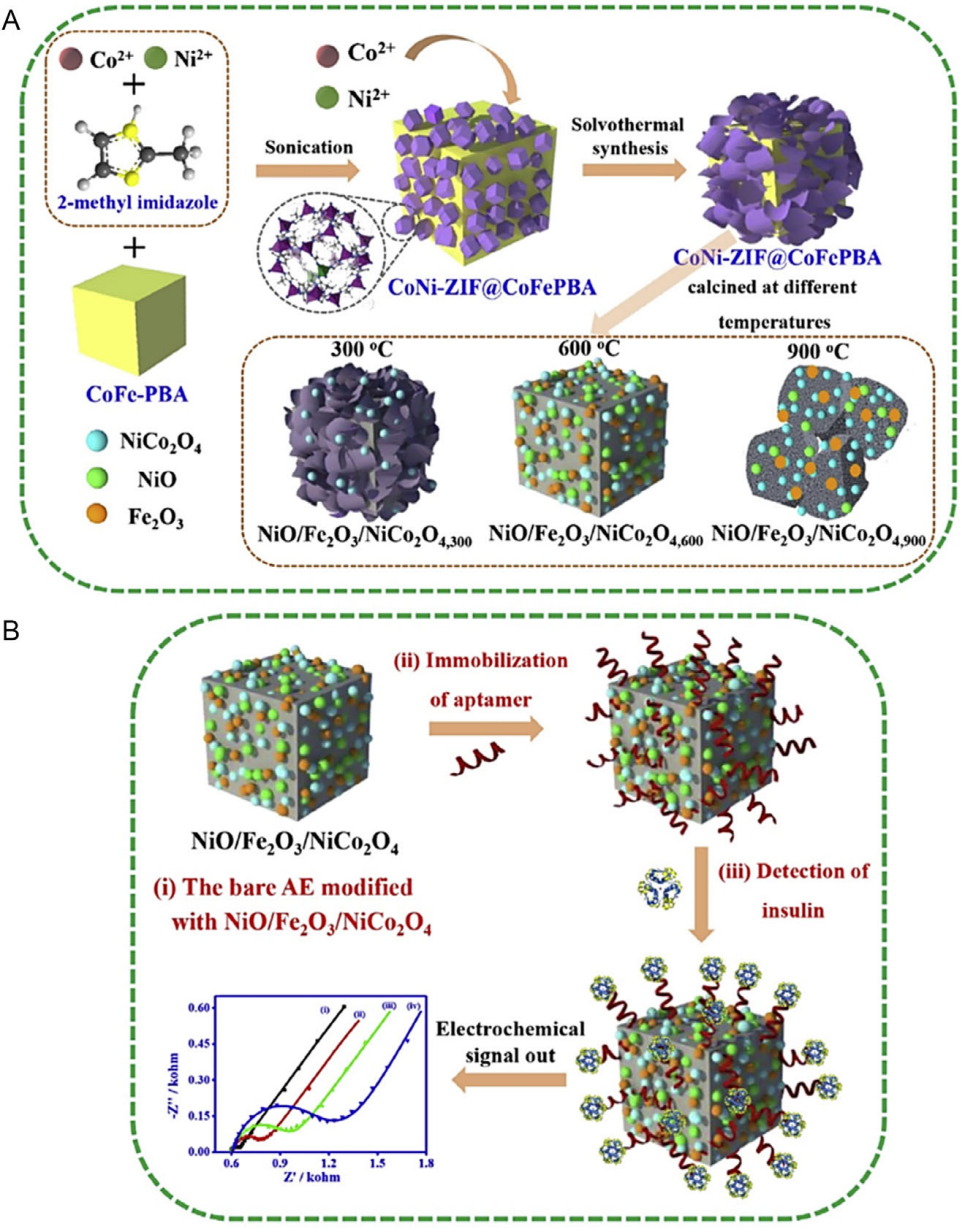
Schematic diagrams of A) the preparation of the series of NiO/Fe_2_O_3_/NiCo_2_O_4_ nanohybrids and B) the development of the NiO/Fe_2_O_3_/NiCo_2_O_4_‐based aptasensor for insulin detection, including (i) coating of electrode with NiO/Fe_2_O_3_/NiCo_2_O_4_ hybrid, (ii) immobilization of insulin aptamer, and (iii) detection of insulin by electrochemical technique. Reproduced with permission.^[^ ^106^ ^]^ Copyright 2020, Elsevier.

An increasingly sophisticated strategy in the development of high‐performance label‐free impedance biosensors involves the construction of hierarchical MOF‐on‐MOF architectures, which are subsequently pyrolyzed to yield mixed‐metal oxide nanohybrids with enhanced electrochemical properties. This templating approach preserves the structural porosity and spatial organization of the original MOFs while introducing synergistic combinations of metal oxides that improve conductivity and catalytic activity. For instance, a NiO/Fe_2_O_3_/NiCo_2_O_4_ nanohybrid derived from a carefully engineered MOF‐on‐MOF precursor has been synthesized (**Figure** **6**).^[^ ^106^ ^]^ This porous, multiphase oxide scaffold exhibited remarkable charge transfer capabilities and surface reactivity, enabling the ultrasensitive detection of insulin at fg/mL concentrations through EIS measurements. This work underscores the potential of MOF‐derived semiconducting nanostructures to serve as powerful transduction interfaces, where even minute interactions with small molecule targets elicit distinct and quantifiable impedance responses.^[^ ^107^ ^]^

Collectively, these studies underscore the dual role of MOFs in label‐free impedance biosensing—as highly tunable platforms for the selective immobilization of biorecognition elements and as active components that enhance electrochemical transduction. The structural regularity and high porosity of MOFs enable efficient capture of target‐specific receptors, while their integration with conductive nanomaterials, such as CNTs, AuNPs, or metal oxides, facilitates rapid electron transfer and amplifies impedance signals. Additionally, the presence of redox‐active metal centers within MOF scaffolds can contribute directly to signal generation, further enhancing detection sensitivity. By leveraging these properties, researchers have demonstrated MOF‐based impedance sensors capable of detecting a wide range of small molecules, including environmental contaminants like pesticides, pharmaceutical residues, and clinically relevant biomarkers, often at nanomolar or even lower concentrations. Looking forward, innovations such as intrinsically conductive MOFs, hierarchically structured MOF derivatives, and multifunctional MOF‐based hybrids are expected to drive the next generation of biosensors.

## 4 Applications

Label‐free EIS biosensors have emerged as a versatile tool across various fields due to their ability to provide rapid, label‐free, and highly sensitive detection. By measuring the electrical impedance changes that occur as a result of biological interactions, these sensors have unlocked new possibilities in diverse areas, including medical diagnostics, environmental monitoring, and food safety. This section will provide a comprehensive overview of the various applications of label‐free impedance biosensors, focusing on their unique advantages and specific use cases within each domain.

### 4.1 Medical Diagnostics

One of the most prominent areas where label‐free EIS biosensors have demonstrated significant potential is in the field of medicine. The capability of these sensors to detect subtle changes in electrical impedance makes them particularly well suited for disease diagnosis and personalized healthcare. Their versatility has led to the development of numerous devices tailored to detect biomarkers, monitor physiological changes, and assist in therapeutic interventions.

#### 4.1.1 Clinical Diagnostics

**Figure 7:**
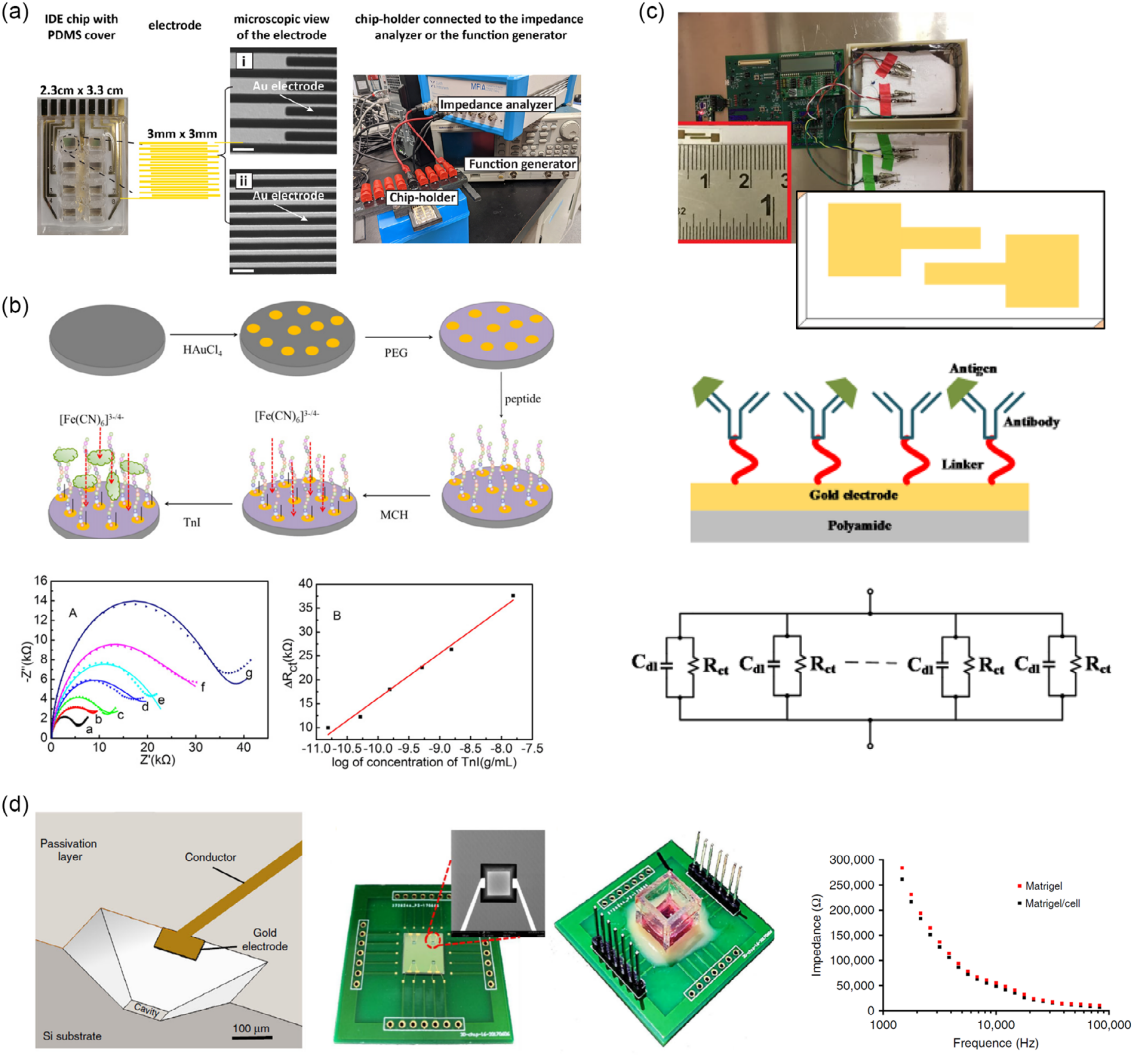
a) Impedimetric sensing for COVID‐19 serology test. Gold micro‐IDEs were used to detect antibodies against SARS‐C0V‐2 based on EIS response. Reproduced with permission.^[^ ^108^ ^]^ Copyright 2022, Elsevier. b) Schematic overview of the peptide‐based EIS biosensor for sensitive detection of cardiac troponin I (TnI). GCE modified with gold nanoparticles (AuNPs) was used as the sensing platform. Reproduced with permission.^[^ ^112^ ^]^ Copyright 2016, Elsevier. c) A four‐channel EIS analyzer module on flexible sensor. Cortisol assay was performed using an ultralow volume (1–3 μL) of perspired human sweat. Reproduced with permission.^[^ ^117^ ^]^ Copyright 2018, Elsevier. d) Silicon‐based multidimensional microgroove impedance sensor (MGIS) for 3D cell trapping and real‐time impedance monitoring. The impedance of 3D cells decreased as the detection frequency increased.^[^ ^121^ ^]^ Open access, Springer Nature, 2020.

Label‐free EIS biosensors are widely used in diagnostics due to their ability to detect disease biomarkers and pathogens with high precision. They have shown remarkable efficacy in diagnosing infectious diseases, including SARS‐CoV‐2,^[^ ^108^, ^109^ ^]^ influenza,^[^ ^110^ ^]^ and tuberculosis.^[^ ^111^ ^]^ During the COVID‐19 pandemic, for instance, an impedimetric biosensor was developed for the rapid detection of antibodies against SARS‐CoV‐2. This sensor utilizes gold interdigitated electrodes (IDEs) configured for EIS, which allows for efficient surface modification with the SARS‐CoV‐2 spike (S) protein (**Figure** **7**a).^[^ ^108^ ^]^ In their evaluation, researchers compared a labeled approach with AuNP‐conjugated secondary antibodies and a direct detection method based on dielectrophoretic force that allowed for the selective capture of anti‐S antibodies from buffer solutions or human sera. In the direct approach, the dielectrophoretic force enriched IgGs near the sensing region, enabling detection within 30 min, faster compared to 3 h with labelled approach. While dielectrophoretic manipulation for direct detection offers promise, it currently demonstrates a one log fold higher LOD compared to a labeled approach (2000 vs. 200 ng mL^−1^), indicating limited sensitivity to detect analytes at low concentrations.

Label‐free EIS biosensors also have been developed to address the urgent need for rapid diagnosis of cardiovascular diseases, particularly myocardial infarction. The detection of cardiac biomarkers such as troponin I (cTnI), troponin T (cTnT), and B‐type natriuretic peptide (BNP) is critical for timely intervention. One innovative biosensor incorporates an AuNP‐modified glassy carbon electrode (GCE) with a peptide probe, self‐assembled on the AuNP surface, enhancing the EIS response and minimizing background noise (Figure 7b).^[^ ^112^ ^]^ The best fit equivalent circuit model used to describe this system is a modified Randles circuit, which incorporates two CPEs connected in parallel with a resistive component, all of which are connected in series *R*_s_ providing a more accurate representation of heterogeneous surface properties introduced by AuNP modification and peptide binding. This configuration achieved a remarkable detection limit of 3.4 pg mL^−1^. Another significant development is an impedance immunosensor designed to detect cTnI using silver nanoparticles (AgNPs) functionalized with 3‐aminopropyltriethoxy silane (APTES).^[^ ^113^ ^]^ This high charge transfer interface equipped with anti‐cTnI antibodies demonstrated a high sensitivity and specificity, with a linear detection range of 20 ng mL^−1^ to 1 μg mL^−1^ and an LOD of 5.5 ng mL^−1^. A notable advancement in the field is the demonstration of multiplexed label‐free detection of cTnI, cTnT, and BNP in human serum at clinically relevant concentrations.^[^ ^114^ ^]^ This biosensor utilizes ZnO‐nanostructured electrodes, which facilitate hemispherical diffusion, a pattern that closely mimics the effects of macromolecular crowding enhancing analyte binding and sensitivity. The improved electron transfer resistance and reduced background signal make this sensor particularly valuable in clinical settings.

Label‐free EIS biosensors also hold promise in oncology, where they are used for real‐time monitoring of cancer cell behaviors, including attachment, spreading, and apoptosis. A notable example is a cell‐based impedance biosensor designed for monitoring the response of MCF‐7 breast cancer cells to the anticancer drug cisplatin.^[^ ^115^ ^]^ The biosensor employs electrical cell‐substrate impedance sensing (ECIS), where increased impedance corresponds to cell attachment and spreading, while a dose‐ and time‐dependent decrease in impedance correlates with drug‐induced apoptosis. Although this assay lacks molecular pathway specificity, this real‐time, noninvasive monitoring capability is crucial for evaluating the efficacy of anticancer therapies.^[^ ^115^, ^116^ ^]^ These studies demonstrate the efficacy of these label‐free EIS biosensors for detecting various biomarkers under laboratory conditions, but significant barriers remain for their real‐world application. These limitations primarily stem from sensitivity and selectivity issues that arise when analyzing complex biological samples.

#### 4.1.2 Healthcare Monitoring

Wearable biosensors have revolutionized continuous health monitoring by providing real‐time tracking of physiological changes without the need for invasive procedures. These sensors are reshaping healthcare management by providing actionable data for real‐time monitoring of physiological and biochemical markers from bedside to even in the comfort of their own homes. Label‐free impedance‐based wearable biosensors are particularly beneficial in chronic disease management, rehabilitation, and fitness monitoring, offering continuous reporting of dynamic health data crucial for personalized care.

One application in wearable healthcare monitoring involves hormone detection. A four‐channel EIS module has been developed for cortisol biosensing in sweat‐based wearable devices. This module, which is integrated with gold‐based flexible chemi‐impedance biosensors, is capable of detecting cortisol concentrations ranging from 1 pg mL^−1^ to 200 ng mL^−1^. Employing a time‐division multiplexing technique allows simultaneous measurements from four sensors, significantly enhancing monitoring accuracy in both synthetic and natural human sweat (Figure 7c).^[^ ^117^ ^]^

Another innovative approach has been developed for continuous glucose monitoring, which involves a battery‐less implantable sensor that operates based on EIS. Designed for subcutaneous implantation, the sensor utilizes a four‐terminal impedance measurement technique to improve accuracy by reducing errors caused by high electrode impedance typically observed in two‐electrode configurations. Tested in pigs, the sensor demonstrated high accuracy with 234 out of 236 measurements falling within a clinically acceptable range, detecting glucose concentrations from 77.4 to 523.8 mg dL^−1^.^[^ ^118^ ^]^ This advancement offers a reliable alternative to traditional blood glucose monitoring methods.

In another study, a multiplexed, noninvasive continuous monitoring platform was engineered for tracking inflammatory markers, particularly C‐reactive protein (CRP) and interleukin‐1β (IL‐1β) as a method to evaluate therapeutic efficacy in patients with inflammatory bowel disease.^[^ ^119^ ^]^ This biosensor construct includes two‐electrode system integrated into a smartwatch‐like wearable device and employs EIS for real‐time monitoring of both CRP and IL‐1β in human sweat. This sensor enabled stable on‐body measurements in a healthy subject cohort for up to 30 h, and performance demonstrated strong Pearson correlation (*r* = 0.99 for IL‐1β and *r* = 0.95 for CRP) with ELISA confirming its reliability and clinical relevance. This strategic integration of EIS biosensors with portable electronic devices exemplifies the shift toward more proactive, preventive, and patient‐centered healthcare monitoring for personalized care.

#### 4.1.3 Drug Screening and Pharmacokinetics

Impedance biosensors play a vital role in the pharmaceutical industry for drug screening and pharmacokinetic studies due to their high sensitivity and their ability to offer real‐time, label‐free monitoring of cellular responses and metabolic changes.^[^ ^120^ ^]^ These biosensors enable monitoring how drugs and their metabolites interact with specific biological targets in real time, making them essential tools for evaluating drug efficacy and tracking pharmacokinetic profiles. For instance, these devices can be coupled with cell‐ and tissue‐specific models to continuously monitor how a drug influences cell behavior (e.g., proliferation) and track their pharmacokinetic profile across various organs. By monitoring kinetics of drug binding to specific receptors or enzymes over time, these sensors offer a cost‐effective and efficient means of assessing drug effects and toxicity, which are critical indicators in drug screening.

Another prominent drug development application is cytotoxicity testing, where label‐free EIS biosensors are used to monitor the viability of cultured cells exposed to potential drug candidates. An advanced 3D microgroove‐based impedance sensor has been developed for noninvasive, high‐throughput monitoring of lung cancer cell viability during anticancer drug evaluation.^[^ ^121^ ^]^ The device traps A549 cancer cells within a Matrigel extracellular matrix within a polymethyl methacrylate chamber fixed onto the microgrooves, which are equipped with gold electrodes that allow for precise EIS‐based monitoring of cell viability and apoptosis (Figure 7d). The cellular response to anticancer drug cisplatin was monitored in this device using EIS, which detected a decreasing impedance response with an increase in cell number. This is because viable cells increase overall conductivity, allowing electrical signal to pass through the network of living cells. A hydrogel‐based diffusion chip integrated with ECIS featuring multiple microelectrodes was developed and used to successfully evaluate the cytotoxic effects of doxorubicin (DOX) and 5‐fluorouracil (5‐FU) on Hela and NIH‐3T3 cell lines, with IC50 values consistent with conventional assays, thereby validating its effectiveness in pharmacological testing.^[^ ^122^ ^]^

### 4.2 Environmental Monitoring

Label‐free EIS biosensors have shown remarkable potential in environmental monitoring due to their high sensitivity, rapid response time, and suitability for on‐site analysis.^[^ ^123^ ^]^ These sensors are particularly valuable for monitoring water quality, air quality, and soil contamination. In this section, we discuss recent advancements and diverse applications of EIS biosensors in environmental monitoring.

#### 4.2.1 Water Quality Assessment

**Figure 8:**
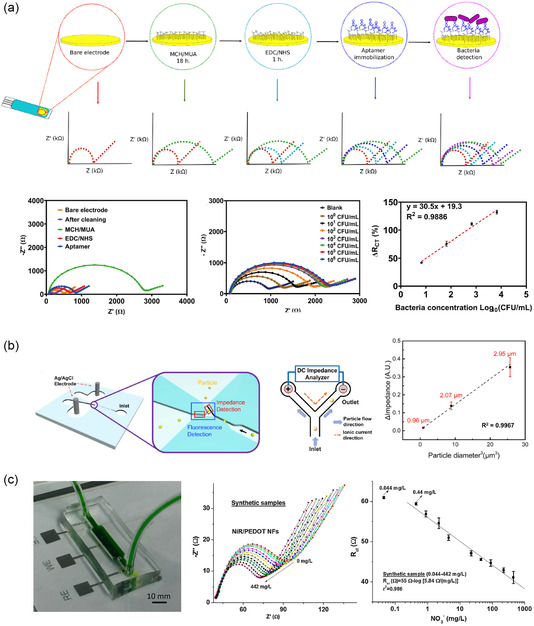
a) Portable impedance‐based aptasensor for *E. coli* detection. Nyquist plots illustrate the electrochemical response at each functionalization step and the impedance spectra for varying *E. coli* concentrations. Reproduced with permission.^[^ ^124^ ^]^ Copyright 2025, Elsevier. b) Bioaerosol monitoring system combining a wet‐cyclone air sampler with a DC impedance microfluidic cytometer. Aerosolized fluorescent microbeads of different diameters were used to evaluate the correlation between the impedance peak amplitude and particle volume. Reproduced with permission.^[^ ^127^ ^]^ Copyright 2021, Elsevier. c) Microfluidic impedimetric sensor using a poly(3,4‐ethylenedioxythiophene) nanofibers (PEDOT‐NFs) and graphene oxide nanosheets for nitrate ion detection. Electrochemical impedance spectra of the NiR/PEDOT NF‐modified electrode were evaluated across different nitrate concentrations. Reproduced with permission.^[^ ^132^ ^]^ Copyright 2017, Elsevier.

Water quality monitoring is crucial for environmental protection as water contamination poses significant risks to human health and ecosystems. Impedance biosensors have been extensively applied in detecting a wide range of water pollutants, including pathogenic microorganisms, heavy metals, and organic contaminants. One prominent application is the detection of microbial pathogens, such as *E. coli* and *Salmonella*, in drinking water and wastewater. For example, a recent study introduced a label‐free impedance sensor specifically designed for the rapid detection of *E. coli* in various matrices, including urine and tap water. This sensor employs a novel aptamer immobilized on a screen‐printed gold electrodes to achieve high sensitivity and specificity.^[^ ^124^ ^]^ It demonstrated an LOD of 1.4 CFU mL^−1^ with a linear detection range from 100 to 10^4^ CFU mL^−1^, completing the analysis within 20 min. Additionally, the sensor displayed minimal cross‐reactivity with nontarget bacteria (e.g., *P. aeruginosa* and *S. aureus*), indicating its robustness for real‐world applications (**Figure** **8**a).

In addition to microbial detection, label‐free EIS biosensors are employed for heavy metal monitoring, addressing critical concerns regarding toxic ions such as Pb^2+^, Cd^2+^, and Hg^2+^.^[^ ^125^ ^]^ One innovative approach utilizes a graphene‐based sensor array for the real‐time detection of Pb^2+^ and Hg^2+^ in flowing tap water, which overcomes fabrication challenges by implementing quality control measures that mitigate device‐to‐device variability. This impedance sensor demonstrates exceptional sensitivity with detection limits as low as 2.5–5 ppb, making it highly suitable for continuous environmental monitoring.^[^ ^126^ ^]^

Similar biosensors also have been developed for detecting organic pollutants, such as pesticides and pharmaceuticals in surface and ground water.^[^ ^127^ ^]^ A notable example is a portable electrochemical EIS platform specifically designed to detect atrazine, a commonly used pesticide.^[^ ^128^ ^]^ The platform integrates a microcontroller and a gold electrode coated with ZnO, significantly enhancing sensitivity through non‐Faradaic EIS. It achieved a remarkably low LOD of 1 fg mL^−1^ and a wide dynamic range from 1 fg mL^−1^ to 10 ng mL^−1^. The system's rapid response time of less than 5 min and minimal sample requirement (20 μL) make it ideal for field‐based pesticide monitoring.

#### 4.2.2 Air Quality Monitoring

Monitoring air quality is essential to detect airborne pathogens and pollutants such as volatile organic compounds and particulate matter, which pose significant health risks. Label‐free EIS biosensors have emerged as a promising technology in this area due to their portability and ability to provide continuous monitoring. A significant development in air quality monitoring involves an integrated bioaerosol monitoring system that combines a DC impedance microfluidic cytometer with a wet‐cyclone air sampler. This system is designed for real‐time detection of airborne bacteria as it efficiently collects bioaerosols, concentrates them into an aqueous solution, and subsequently analyzes the sample using a microfluidic cytometer for DC impedance measurements (Figure 8b).^[^ ^129^ ^]^ This biosensor was able to differentiate between dust, live *E. coli*, and dead *E. coli* using fluorescence staining, achieving an overall detection efficiency of 24.59%.

Another noteworthy advancement involves detecting toxic vapor concentrations, especially in industrial environments.^[^ ^130^ ^]^ For instance, a selective vapor sensor based on layered black phosphorus was developed to detect methanol.^[^ ^131^ ^]^ Using EIS as the detection method, the sensor shows a linear response to methanol vapor concentrations ranging from 380 to 1900 ppm, with a low detection limit of 28 ppm, significantly lower than the safety threshold of 200 ppm. This sensor demonstrated high specificity for methanol, maintaining stability for over 20 days, making it suitable for industrial safety applications.

#### 4.2.3 Soil Monitoring

Monitoring soil contamination is essential for agricultural sustainability and environmental safety. Label‐free EIS biosensors have demonstrated substantial potential in detecting soil pollutants, such as pesticides, heavy metals, and nitrates. For example, a microfluidic impedimetric sensor has been developed for nitrate detection in soil, which employs a composite of graphene oxide and poly(3,4‐ethylenedioxythiophene) nanofibers (PEDOT‐NFs) that increase the electrochemical surface area and electron transfer rate (Figure 8c).^[^ ^132^ ^]^ Nitrate reductase (NiR) enzyme immobilized on this composite facilitates the catalytic conversion of nitrate to nitrite enabling sensitive detection. The sensor achieved a detection limit of 0.135 mg L^−1^ and demonstrated strong selectivity against interfering ions, and it proved reliable when applied to real soil samples.

### 4.3 Food Safety

Impedance biosensors have become invaluable in food safety applications due to their rapid detection, high sensitivity, and potential for on‐site analysis. Their capabilities have been harnessed for the detection of pathogens and allergens, as well as for quality control and shelf life assessment.

#### 4.3.1 Pathogen Detection

**Figure 9:**
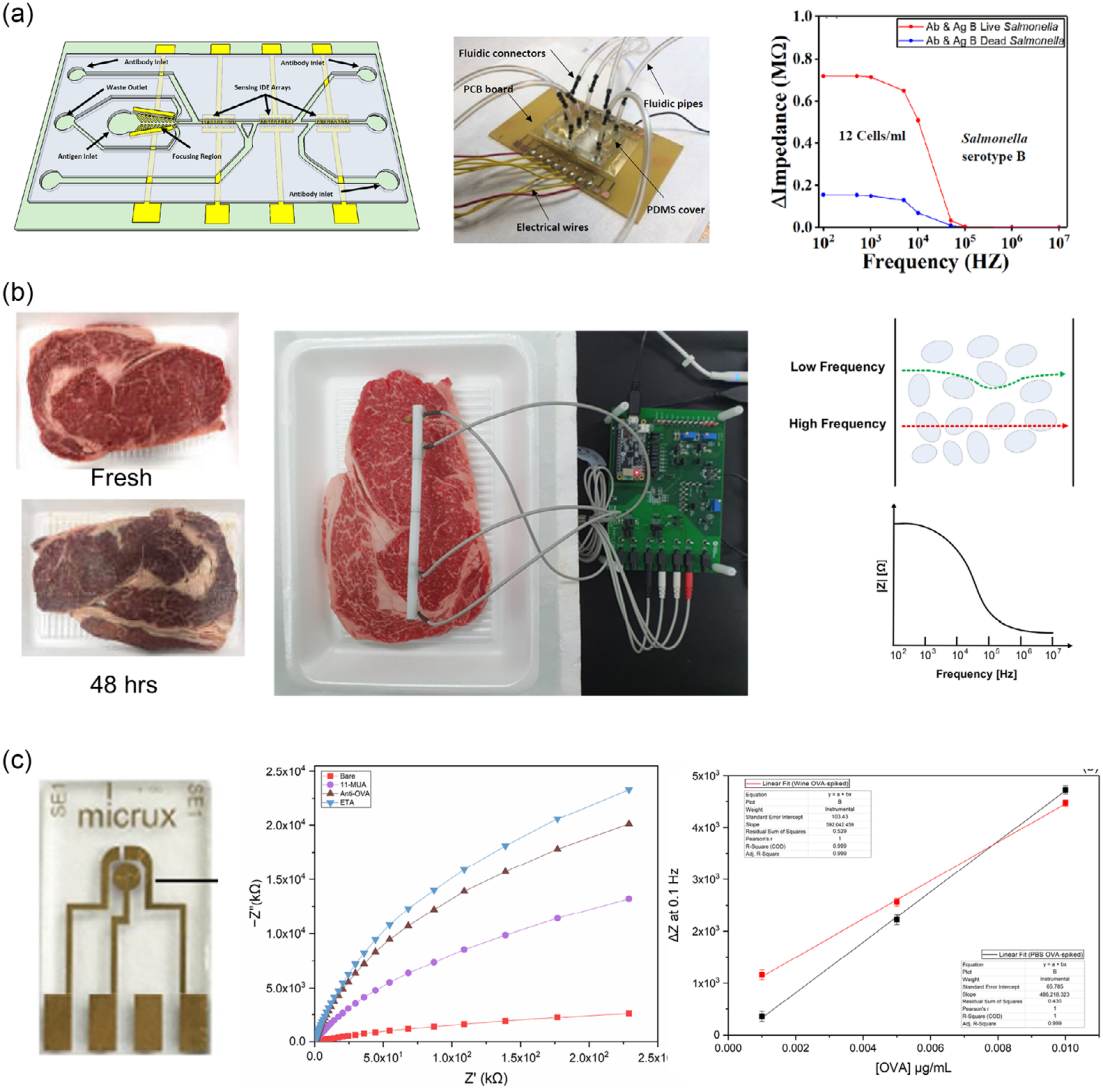
a) Impedance‐based biosensor for rapid, simultaneous detection of *Salmonella* serotypes B, D, and E. The sensor successfully differentiated live and dead *Salmonella* serotype B at 12 cells mL^−1^, highlighting its capability to identify bacterial viability.Reproduced with permission.^[^ ^134^ ^]^ Copyright 2018, Springer Nature. b) EIS‐based approach for meat freshness assessment using image classification for nondestructive, cost‐effective analysis. At low frequencies, current is restricted by the high resistance of cell membranes, while at high frequencies, it penetrates both extracellular and intracellular spaces.^[^ ^143^ ^]^ Open access, MDPI, 2021. c) Impedance‐based immunosensor for ovalbumin detection in white wine. Gold thin‐film electrodes were sequentially modified with 11‐MUA, NHS/EDC, anti‐OVA polyclonal antibodies, and ETA. The resulting sensor was tested with varying concentrations of OVA.^[^ ^137^ ^]^ Open access, MDPI, 2023.

Foodborne pathogens are a major public health concern, and the rapid detection of these microorganisms is essential for ensuring food safety. Label‐free EIS biosensors have demonstrated significant potential in identifying bacterial pathogens, including *Salmonella*, *E.* *coli O157:H7*, and *Listeria monocytogenes*.^[^ ^133^ ^]^ For instance, an integrated impedance biosensor platform was developed for detecting *Salmonella* serotypes B, D, and E in poultry products.^[^ ^134^ ^]^ This platform utilizes a dielectrophoretic focusing region to concentrate bacterial cells, followed by three IDE arrays functionalized with anti‐*Salmonella* antibodies. The biosensor achieved a detection limit of 8 cells mL^−1^ within 45 min and was capable of distinguishing live from dead cells, demonstrating high selectivity (**Figure** **9**a).

Impedance biosensors also have been developed to detect viral pathogens. An ultrasensitive DNA aptasensor was developed to detect noroviruses (MNV and HuNoV) using a high‐affinity aptamer immobilized on a gold nanoparticle‐modified screen‐printed carbon electrode.^[^ ^135^ ^]^ EIS is utilized to detect viral binding by measuring changes in *R*_ct_, which increases upon virus attachment. The sensor demonstrated a very low detection limit of 10 aM with high specificity, distinguishing noroviruses from other viral and protein interferences.

#### 4.3.2 Allergen Detection

Food allergens also pose a significant public health concern, and the demand for rapid, accurate, and sensitive detection methods has been growing. Impedance biosensors have shown great promise in detecting allergens due to their high specificity and quick response, making them suitable for use in food production and safety monitoring.^[^ ^136^ ^]^ A non‐Faradaic impedimetric immunosensor was developed to detect ovalbumin in white wine, addressing concerns regarding egg allergens.^[^ ^137^ ^]^ The sensor uses EIS to monitor antigen–antibody interactions without requiring a redox probe, thereby simplifying the detection process. The immunosensor exhibited a detection limit of 0.20 μg mL^−1^, complying with international safety guidelines. Notably, it demonstrated high sensitivity with a short response time of 15 min and stability over 2 weeks, making it an effective tool for real‐time allergen monitoring in the wine industry (Figure 9c).

In addition to detecting specific allergens like ovalbumin, impedance biosensors have been developed to detect proteins associated with common food allergies, including gluten, casein, and peanut proteins. A silicon‐based electrochemical immunosensor for the rapid detection of IgG antibodies linked to peanut and hazelnut allergens was created using EIS combined with copper‐free click chemistry.^[^ ^138^ ^]^ The device demonstrated high specificity by immobilizing allergen‐specific peptides on gold electrodes, achieving reliable detection of IgG antibodies in diluted serum samples. The microchip was tested with sera from rabbits immunized with peanut and hazelnut proteins, showing reliable detection within dilution ranges from 1:500 000 (0.0002%) to 1:50 000 (0.002%). This technology represents a significant advancement in food allergy diagnostics by offering enhanced portability, rapid results, and high accuracy, thereby addressing the limitations of traditional ELISA‐based methods.

Another study reported an EIS‐based device engineered on a galvanically/electroless deposited silicon substrate over an aluminum alloy for the detection of Ara h 1, one of the most clinically relevant peanut proteins.^[^ ^139^ ^]^ The impedance characteristics of this sensor were fit with an equivalent circuit consisting of three RC loops connected in series each representing specific surface behavior. The sensor demonstrated highly sensitive, concentration‐dependent changes in *R*_ct_ yielding a detection limit as low as 4 ng mL^−1^, substantially below the reported 1–100 μg mL^−1^ allergenic threshold in food. Another notable advancement is the development of mast cell‐based electrochemical biosensors. Jiang et al. reported a gold nanoparticle‐based biosensor for the quantification of shrimp tropomyosin (Pen a 1), the major shrimp allergen.^[^ ^140^ ^]^ This sensor utilized IgE‐mediated mast cell sensitization to generate impedance signals upon allergen exposure, achieving a detection limit as low as 0.15 μg mL^−1^. While we have focused on selective examples, a recent review highlights the versatility of these biosensors as transformative platforms for real‐time and POC monitoring throughout the food supply chain, enabling sensitive and selective detection of common food allergens.^[^ ^141^ ^]^

#### 4.3.3 Quality Control and Shelf Life Assessment

Maintaining food quality and accurately assessing shelf life are crucial for the food industry to ensure safety and reduce economic losses. Impedance biosensors have proven particularly valuable in this context due to their ability to detect biochemical changes associated with food spoilage in a rapid, noninvasive, and on‐site manner.^[^ ^142^ ^]^ One recent study for food quality assessment involved combining EIS data with image classification to enhance the accuracy of meat freshness detection.^[^ ^143^ ^]^ The system developed for this purpose integrated impedance measurements with visual data analysis, achieving a prediction accuracy of up to 85% for freshness classification compared to 56.7% when using image data alone. This dual‐modality approach highlights how combining electrical and visual cues significantly improves the reliability of freshness evaluation (Figure 9b).

Another study focused on monitoring the spoilage of salmon using EIS and time‐domain terahertz spectroscopy to assess freshness nondestructively.^[^ ^144^ ^]^ This system provided a comprehensive assessment of spoilage progression, identifying four distinct freshness stages: fresh, semifresh, semideteriorated, and deteriorated. The ECIS system detected critical frequency shifts and phase changes correlating with spoilage, demonstrating greater sensitivity in the early stages compared to traditional methods. This novel approach not only ensures accurate freshness monitoring but also offers real‐time, noninvasive assessment capabilities, making it suitable for routine food quality control.

A major challenge to food safety is mycotoxin contamination during storage, particularly in commodities such as cereals, coffee, dried fruits, and meat sourced from contaminated feeds. Ochratoxin A (OTA), a toxin produced by *Aspergillus* and *Penicillium* species, is among the most widespread storage‐associated toxins and has been classified as a potential human carcinogen. To address this issue, Norouzi et al. developed a nanocomposite EIS sensor based on ionic liquid‐graphene nanosheets decorated with gold nanoparticles for the sensitive detection of OTA.^[^ ^145^ ^]^ The platform exhibited remarkable analytical performance, with a detection limit of 2.2 × 10^−10^ m. Such developments illustrate the potential of electrochemical impedance‐based approaches in monitoring toxin accumulation during storage, thereby enabling early intervention to ensure food safety and compliance with regulatory standards.

### 4.4 Commercialization Challenges

Despite remarkable progress in label‐free EIS biosensors, their translation into real‐world applications has remained limited. The challenges to commercialization are highly application‐specific and extend beyond sensitivity and selectivity, encompassing issues of robustness, reproducibility, and integration with existing infrastructures. In clinical diagnostics, the foremost obstacle is sensor reliability in complex biofluids, such as blood, serum, and saliva, where nonspecific adsorption and electrode fouling reduce analytical performance. Reproducible sensor fabrication, biorecognition element stability, and rigorous clinical validation remain critical unmet needs. Furthermore, successful deployment requires user‐friendly device formats that can operate in POC settings and comply with strict regulatory frameworks governing diagnostic technologies. For environmental monitoring, the primary barriers relate to robustness and stability under diverse and often harsh field conditions. Variability in pH, ionic strength, and contaminant load can compromise impedance readouts. Additionally, regulatory limits for pollutants demand extremely low detection thresholds, which must be achieved without sacrificing portability or cost‐effectiveness—an essential requirement for wide adoption in resource‐limited settings. In the area of food safety, the complexity and heterogeneity of food matrices present formidable challenges, as fats, proteins, and other components may obscure or interfere with specific analyte signals. Overcoming these issues requires standardized sample preparation methods and multiplexing capability to simultaneously screen for pathogens, toxins, or allergens. Cost‐per‐test, rapid turnaround, and scalability of production are also decisive factors for successful adoption in industrial quality control workflows. Taken together, these hurdles underscore that commercialization of label‐free EIS biosensors will require not only advances in materials and biorecognition chemistry but also system‐level solutions addressing manufacturability, regulatory approval, and seamless integration into existing infrastructures.

## 5 Conclusion and Future Outlook

From the discussion above, it is clear that remarkable progress has been made in the development and application of impedance‐based label‐free biosensors over the past few decades. There have been tremendous efforts to develop and integrate novel and robust nano‐and micromaterials, system architectures, and biorecognition elements capable of maintaining high specificity in a complex biological environment. While development of more sensitive biorecognition molecules and their immobilization on the surface of electrodes have helped to enhance detection of impedance changes, advances in nanomaterials and sensor architecture have also significantly increased system sensitivity and miniaturization. Despite these advancements, the field of label‐free biosensors has not yet achieved full commercial translation because several formidable challenges persist, which will require further refinement to overcome.

The most daunting issue is the Debye length screening, which limits the detection of biorecognition events beyond few nanometers from the electrode surface. While nanoengineered sensor architectures integrating high‐affinity biorecognition elements such as aptamers have been shown to mitigate these effects and improve sensitivity, they introduce their own challenges.^[^ ^101^ ^]^ Moreover, these biosensing platforms cannot be adapted for integration of larger elements like antibodies. Our group has recently developed a novel method that integrates a thin functional polymeric layer with biorecognition elements to shift EDL outward, thereby enhancing sensitivity in label‐free biosensing.^[^ ^146^ ^]^ This strategy can be broadly applied to all biorecoginition systems to overcome charge‐screening effects that hinder conventional electrochemical sensors. Additionally, approaches including surface engineering electrodes using nanostructures and advanced polymeric coatings to confine Debye volume and disrupting EDL through external perturbations are currently being explored to overcome these limitations.^[^ ^13^ ^]^

Susceptibility of electrochemical interfaces to fouling is particularly problematic for EIS‐based biosensors, as even small changes in the interfacial properties, such as those caused by nonspecific surface binding interactions, can lead to pronounced changes in impedance response, resulting in reduced selectivity and unreliable sensor performance. Although different antifouling‐based surface chemical modifications have been reported to date, slower diffusion rates impact binding kinetics and increase detection time restricting their potential for POC applications. Additionally, long‐term stability and resistance to fouling in real samples is critical for practical applications. We have developed a porous nanocomposite‐based antifouling coating for electrochemical biosensors that is both electroconductive and prevents fouling.^[^ ^7^, ^147^ ^]^ This approach has been used successfully for affinity‐based electrochemical detection of several analytes in various sample matrices, even whole blood, using a labeled (indirect) biosensor approach.^[^ ^147^, ^148^ ^]^ However, the same antifouling coating has been adapted for label‐free sensing by applying this conductive antifouling coating to an aptamer‐based biosensor for cortisol detection, highlighting its versatility and effectiveness.^[^ ^53^ ^]^ But the impact of these coatings on impedance response and overall sensitivity for direct detection remains to be fully evaluated.

Impedance in electrochemical systems can change significantly over time due to dynamic processes occurring at the electrode surface and within the aqueous electrolytic solution. Signal drift is a well‐documented issue in these biosensors, which significantly impacts reliability and reproducibility underscoring the need for integrating advanced AI/ML models for data interpretation. This also could offer insights into optimizing measurement conditions so as to minimize drift and improve signal‐to‐noise ratio. In addition, time‐dependent impedance characterization can provide critical insights for a deeper understanding of both the EIS technique and biorecoginition events underlying impedance changes at the electrode/electrolyte interfaces, which is essential for optimizing sensor design and achieving reliable sensor performance.

Although these technological improvements push the limits of sensitivity and selectivity in label‐free EIS biosensors, it is important to ensure their reliable and robust performance in real‐world applications. The expectation is that these sensors must deliver reproducible results between multiple tests with real samples. This requires rigorous standarization of testing protocols, inclusion of appropriate negative and positive controls, and implementation of calibration procedures consistently by all developers across the field to ensure data from different studies and between different research groups are directly comparable. Establishing universal standards and quality control practices will not only improve biosensor reliability and reproducibility but also make it easier to compare and benchmark technological advancements in the development of label‐free EIS biosensors. Standardized evaluation will accelerate progress by enabling researchers to compare performance, understand limitations, and develop sensors best suited for each specific application. This will ultimately facilitate the translation of these biosensor technologies toward practical utility. Overall, while the field has advanced considerably, overcoming these technical barriers remains essential for realizing the full potential of label‐free EIS biosensors in real‐world applications.

## Conflict of Interest

D.E.I. is a founder, holds equity, and is a board member at StataDx Inc. P.J. is a co‐founder, and holds equity at StataDx Inc. N.R.S., J.C.L., P.J., and D.E.I., are listed as inventors on patents describing label‐free electrochemical technology. J.R. declare no conflict of interest.
